# Supplementary material for: Geographic variation in COVID-19 vulnerability by legal immigration status in California: a prepandemic cross-sectional study
Source: BMJ Open. 2022 May 23;12(5):e054331. doi: 10.1136/bmjopen-2021-054331 (PMC9130646; doi:10.1136/bmjopen-2021-054331)
Supplement: Author's manuscript [file bmjopen-2021-054331.draft_revisions.pdf]

BMJ Open

BMJ Open is committed to open peer review. As part of this commitment we make the peer review history of every article we publish publicly available.

When an article is published we post the peer reviewers' comments and the authors' responses online. We also post the versions of the paper that were used during peer review. These are the versions that the peer review comments apply to.

The versions of the paper that follow are the versions that were submitted during the peer review process. They are not the versions of record or the final published versions. They should not be cited or distributed as the published version of this manuscript.

BMJ Open is an open access journal and the full, final, typeset and author-corrected version of record of the manuscript is available on our site with no access controls, subscription charges or pay-per-view fees (http://bmjopen.bmj.com).

If you have any questions on BMJ Open's open peer review process please email info.bmjopen@bmj.com

BMJ Open

Ecological Study of COVID-19 Vulnerability by Legal Immigration Status in California

Journal:	*BMJ Open*
Manuscript ID	bmjopen-2021-054331
Article Type:	Original research
Date Submitted by the Author:	10-Jun-2021
Complete List of Authors:	Sohn, Heeju; Emory University, Sociology<br>Aqua, Jasmine; Emory University, Rollins School of Public Health, Epidemiology
Keywords:	COVID-19, Public health < INFECTIOUS DISEASES, Epidemiology < TROPICAL MEDICINE

SCHOLARONE™
Manuscripts

I, the Submitting Author has the right to grant and does grant on behalf of all authors of the Work (as defined in the below author licence), an exclusive licence and/or a non-exclusive licence for contributions from authors who are: i) UK Crown employees; ii) where BMJ has agreed a CC-BY licence shall apply, and/or iii) in accordance with the terms applicable for US Federal Government officers or employees acting as part of their official duties; on a worldwide, perpetual, irrevocable, royalty-free basis to BMJ Publishing Group Ltd ("BMJ") its licensees and where the relevant Journal is co-owned by BMJ to the co-owners of the Journal, to publish the Work in this journal and any other BMJ products and to exploit all rights, as set out in our licence.

The Submitting Author accepts and understands that any supply made under these terms is made by BMJ to the Submitting Author unless you are acting as an employee on behalf of your employer or a postgraduate student of an affiliated institution which is paying any applicable article publishing charge ("APC") for Open Access articles. Where the Submitting Author wishes to make the Work available on an Open Access basis (and intends to pay the relevant APC), the terms of reuse of such Open Access shall be governed by a Creative Commons licence – details of these licences and which Creative Commons licence will apply to this Work are set out in our licence referred to above.

Other than as permitted in any relevant BMJ Author's Self Archiving Policies, I confirm this Work has not been accepted for publication elsewhere, is not being considered for publication elsewhere and does not duplicate material already published. I confirm all authors consent to publication of this Work and authorise the granting of this licence.

For peer review only

Ecological Study of COVID-19 Vulnerability by Legal Immigration Status in California

Heeju Sohn, Ph.D.*

Department of Sociology

Emory University

Jasmine Ko Aqua, M.P.H.

Department of Epidemiology

Rollins School of Public Health

Emory University

* Corresponding author

Funding Information

This project was supported by grant number R00HD096322 from the Eunice Kennedy Shriver National Institute of Child Health and Human Development (NICHD).

For peer review only - http://bmjopen.bmj.com/site/about/guidelines.xhtml

Ecological Study of COVID-19 Vulnerability by Legal Immigration Status in California

For peer review only - http://bmjopen.bmj.com/site/about/guidelines.xhtml

Contributorship statement

Heeju Sohn acquired funding and access to the restricted data, lead the conceptualization and the analysis, and contributed to writing and editing the manuscript. Jasmine Aqua contributed to the methodology, the literature view, and writing and editing the manuscript.

Data sharing statement

The restricted version of the California Health Interview Survey (CHIS) is available through the Data Access Center at UCLA's Center for Health Policy Research. https://healthpolicy.ucla.edu/

For peer review only

Abstract

Objective: To quantify COVID-19 vulnerabilities for Californian residents by their legal immigration status and place of residence.

Design: Secondary data analysis

Data: All adult respondents in the restricted version of the California Health Interview Survey (2014-2019, n = 189,754)

Outcome measure: Relative Social Vulnerability Indices for COVID-19 by legal immigration status and Census region across six domains: socioeconomic vulnerability; demography and disability; minority status and language barriers; high housing density; epidemiological risk; and access to care.

Results: Undocumented immigrants living in Los Angeles and Orange Counties have exceptionally high vulnerabilities due to low socioeconomic status, high language barriers, high housing density, and low access to care. San Joaquin Valley is home to both vulnerable immigrant groups and a native population with the highest demographic and epidemiological risk for severe COVID-19.

Conclusion: Interventions to mitigate public health crises must explicitly consider immigrants' dual disadvantage form social vulnerability and exclusionary state and federal safety-net policies.

Keywords: COVID-19 Vulnerability, Immigrants, United States

For peer review only

For peer review only - http://bmjopen.bmj.com/site/about/guidelines.xhtml

Strengths and limitations of this study

1. We adapted the CDC's Social Vulnerability Index (SVI) to quantify immigrants' vulnerability to COVID-19 by their legal immigration status and their geographic region of residence in California.
2. Our analysis used the California Health Interview Survey (2014-2019, n = 189,754) which contains direct measurements of immigrants' legal status as well as detailed socioeconomic and health information.
3. The data covers the years 2014 to 2019 and vulnerability indices may diverge from the pandemic's peak in 2020.
4. Vulnerability indices are relative measures among California's 50 immigrant status-region groups and cannot be generalized to the broader national population.

For peer review only

The novel coronavirus, severe acute respiratory syndrome coronavirus 2 (SARS-CoV-2), has spread across all parts of the United States, exacerbating entrenched social and health inequalities in its wake. This article uses sensitive immigration and geographic information from the restricted data in the California Health Interview Survey (CHIS) to quantify underlying socioeconomic, demographic, and epidemiological vulnerabilities to COVID-19 by legal immigration status in California's ten census regions.

Prior research on immigrants' resources and health suggests that their vulnerability to COVID-19 may be higher than the native U.S. population [1]. Many immigrants work in occupations that required in-person work throughout the pandemic [2,3], and they live in larger households making isolation difficult [4]. Higher prevalence of health conditions such as obesity, asthma, and diabetes among socioeconomically disadvantaged groups [5,6] suggests that immigrants may also have a higher risk for severe COVID-19 outcomes.

At the same time, immigrants comprise a large and diverse group [7,8] in which some sub-groups have high levels of education and income, whereas other sub-groups have high rates of poverty and economic insecurity. Unequal distribution of health care resources across geographic regions and residential segregation may also contribute to inequities in COVID-19 mortality within immigrant communities [9]. Yet, systematic and precise information on immigrants' vulnerabilities is absent from policymaking due to the lack of detailed immigration information in population-representative health surveys.

Federal policies dictate immigrants' access to federally funded health care services and safety-net programs [10,11] based on their immigration status. Undocumented immigrants face especially high risk for adverse COVID-19 outcomes. Not only do they share COVID-19 risk factors with other disadvantaged populations, but they also have greater barriers in accessing health care and safety-net programs [12]. The recent Coronavirus Aid, Relief, and Economic Security Act explicitly barred undocumented immigrants

For peer review only - http://bmjopen.bmj.com/site/about/guidelines.xhtml

from receiving financial relief, continuing the long-standing policy of barring undocumented immigrants from cash assistance [13]. Legally present visa-holders and immigrants who have permission to live and work in the U.S. also have limited access to resources they can turn to during crises. The 1996 Personal Responsibility and Work Opportunity Reconciliation Act restricted legal immigrants' eligibility for federally funded safety-net programs, [10] and U.S. Citizenship and Immigration Services (USCIS) issued guidance at the beginning of the pandemic that legal immigrants could be denied citizenship or permanent residency for receiving an expanded range of eligible public health care benefits[14,15].

Policies at the state and local levels can support inclusive public health programs and outreach to address their immigrant communities' specific needs [16,17]. Some localities have expanded health care services to undocumented immigrant children and low-income pregnant women, allowed for in-state tuition and financial aid for undocumented students, and issued government identification to all residents [18]. Inclusive policies facilitate schooling and employment [19] for vulnerable immigrant groups and have been linked to better health outcomes [20]. Conversely, exclusionary policies such as mandating the use of E-Verify, an electronic database of immigrants' work authorization, or barring states from issuing drivers' licenses or granting college admission to undocumented immigrants aim to create obstacles for those who do not have legal status [18,21]. Localities that coordinate with immigration enforcement also deter many immigrants and their families from seeking help regardless of their citizenship status [22,23].

This article identifies opportunities for local- and community-level interventions that can address the immigrants' unique challenges. Fractured policies that stratify people by immigration status stymies efforts that aim to mitigate the effects of the COVID-19 pandemic for all.

For peer review only - http://bmjopen.bmj.com/site/about/guidelines.xhtml

Methods

Data Source

We used the 2014-2019 survey data from CHIS, a collaborative data collection between UCLA's Center for Health Policy Research, the California Department of Public Health, and the Department of Health Care Service [24]1/1/0001 12:00:00 AM. The CHIS is a large, annual random-digit telephone survey of public health and health care access issues in California and is one of few representative surveys of this scale that collects sensitive information on immigrant documentation status.

Study Population

Our analysis included 189,754 adult survey respondents 18 years or older and used individual weights to account for sampling design. The CHIS imputed missing values for almost all variables in their surveys using random selection or hot deck imputation used in Census-published datasets.

Patient and public Involvement statement

The article presents analyses of secondary survey data, and no patients were involved in the study.

Documentation Status

We categorized respondents by their nativity and legal immigration status: U.S.-born natives, naturalized citizens, legal permanent residents (LPR), documented temporary visa holders, and undocumented immigrants. The first three categories were determined directly for the entire study period 2014-2019 through a series of citizenship and immigration questions. All respondents answered whether they were born in the United States. If the response was no, they indicated whether they were naturalized citizens. Respondents who were not U.S. citizens were then asked whether they were LPRs. Questions that can differentiate undocumented immigrants from non-LPRs with valid U.S. visas are sensitive and were only asked 2015-2016. The large majority (97.71%) of our analysis sample had direct

For peer review only - http://bmjopen.bmj.com/site/about/guidelines.xhtml

information on immigration status, including whether they were undocumented or living in the U.S. on valid visas. The remaining 2.29 percent of our sample with missing immigration status data were non-LPRs in the years 2014, 2017, 2018, and 2019 when CHIS did not ask specific details on visa status. We used a multiple imputation procedure to impute missing values based on the relationships between sociodemographic characteristics and documentation status derived from the complete survey years in 2015 and 2016 [25,26]. We included age, age-squared, sex, educational attainment, country of origin, family type, English proficiency, years lived in the U.S., federal poverty level, and geographic location in our multiple imputation procedure[27].

COVID-19 Vulnerability Index

We adapted the validated U.S. Centers for Disease Control and Prevention (CDC) 's Social Vulnerability Index (SVI)[28] to develop a COVID-19 vulnerability index. We modified the CDC SVI based on the variables available in our dataset and expanded the index to include additional factors critical to the COVID-19 pandemic [29]. Overall, we considered 21 factors across six domains in our COVID-19 vulnerability index (Table 1).

[Table 1]

Domain 1 – Socioeconomic Vulnerability: This domain captures the disproportionate crisis vulnerability associated with economic disadvantage. Households living below the poverty line face increased COVID-19 vulnerability due to structural health inequities and disproportionate distribution of underlying comorbidities[30,31]. Individuals with higher educational attainment have greater access to and may better adapt to COVID-19 risk communications and health messaging[32].

Domain 2 - Demographic Vulnerability & Disability: This domain captures the increased danger that vulnerable demographic groups face in disaster situations such as the COVID-19 pandemic. Older adults are at greater risk of requiring hospitalization or dying if diagnosed with COVID-19, and single parents

For peer review only - http://bmjopen.bmj.com/site/about/guidelines.xhtml

and individuals with disabilities may experience additional stressors of the pandemic [33]. The pandemic has been particularly challenging for single-parent households, where only one parent is available for multiple responsibilities that may include working extra shifts, caring for a sick family member, or supervising online schooling.

Domain 3 - Minority Status & Language Barriers: This domain captures minority and marginalized populations' disproportionate vulnerability. About 33 percent of US-born and 60 percent of the foreign-born population in California self-reported as non-white[34], and they may encounter more racialized discrimination in health care settings than their white counterparts [35]. Limited English proficiency can be a barrier to accessing health services and understanding COVID-19 health messaging; recent studies linked low English proficiency with an increased risk of COVID-19 [32].

Domain 4 - High Housing Density: We included density factors associated with an increased risk of SARS-CoV-2 transmission: the proportion of respondents who live in a multi-family or mobile house, the proportion of respondents who live in an urban or metropolitan area, and the proportion of households with three or more adults [36].

Domain 5 - Epidemiological Risk Factors: This domain captures the medical and epidemiologic risk factors associated with COVID-19 infection and its adverse outcomes. The medical risk factors for severe COVID-19 in this domain include cardiovascular conditions (high blood pressure and heart disease), respiratory conditions (asthma and smoking), obesity, and diabetes [33]. Epidemiologic risk factors included occupations with a high risk of COVID-19 exposure. We used the O* NET's Work Surveys to identify high-risk occupations and cross-referenced them with California's Executive Order N-33-20 that defined essential workers. We harmonized the occupation categories with CHIS and assigned occupations in healthcare, service, transportation, construction, and extraction in the high-risk category.

For peer review only - http://bmjopen.bmj.com/site/about/guidelines.xhtml

Domain 6 - Low Access to Health Care: This domain encapsulates the additional vulnerability that health care barriers, such as the lack of health insurance, add during a widespread health crisis. Concerns about the cost of testing and treatment and uncertainty around where to seek medical attention lead to delayed patient care and disrupt our ability to control epidemics [32].

We constructed a vulnerability index for each of the six domains by immigration status intersected with census region (5 immigrant groups x 10 regions = 50 immigrant-region groups). First, we estimated groups' proportions in the high vulnerability category for each of the 21 factors. Second, we averaged the proportions across factors within each domain. Third, we ranked immigrant status-region groups from the group with the highest proportion in the vulnerable category to the lowest. We then assigned a percentile rank using the following equation: Percentile Rank = (rank – 1)/(N-1) where N equals 50 and represents the total number of immigrant status-region groups. A higher percentile indicates greater relative vulnerability. Our approach is the same as the method used by Acharya and Porwal [29], and Flanagan and colleagues [37].

Results

Table 2 reports vulnerability indices in six domains for five immigrant groups living in California's ten census regions. Indices range from 0 (least vulnerable) to 1 (most vulnerable) and represent the relative vulnerability within 50 immigrant-region groups.

[Table 2]

Undocumented immigrants have high vulnerability due to low socioeconomic status, concentration of minorities and language barriers, and low access to care across the entire state. Undocumented immigrants living in the Inland Empire and San Joaquin Valley have the highest socioeconomic vulnerability (1.00 and 0.98, respectively). In contrast, vulnerability due to minority status and language

For peer review only - http://bmjopen.bmj.com/site/about/guidelines.xhtml

barriers is the highest among undocumented immigrants in Orange County (0.98) and Central Coast (1.00).

Naturalized citizens and US-born natives share similar vulnerability profiles across the ten regions, and unlike non-citizen immigrants, their sources of vulnerability are predominantly from demographic composition and disability. They also score high in vulnerability from epidemiological COVID-19 risk factors, especially in the North Coast and the San Joaquin Valleys.

The wide range of vulnerability indices across California's regions reflects documented temporary visa holders' socioeconomic and demographic diversity. Documented visa holders living in the San Francisco-Bay Area are among the least vulnerable—they are the most socioeconomically and demographically advantaged (indices of 0.0) with low epidemiological risk for COVID-19. Conversely, documented visa-holders living in Southern San Joaquin Valley have a high socioeconomic vulnerability (0.939) and low access to health care (0.0.8).

Vulnerability due to high housing density is concentrated among non-citizen immigrants, including LPRs in Southern California—Los Angeles County, Orange County, and San Diego-Imperial—and is likely linked to high housing costs in these regions. San Joaquin Valley is home to vulnerable non-citizen immigrants, including LPRs, due to their low socioeconomic status, high minority populations, and language barriers.

Table 2 also reports the overall vulnerability that combines all six domains, and the last column in the table indicates its ranking among the 50 immigrant status-region groups. Undocumented immigrants living in Southern California had the highest overall vulnerability. US-born natives and documented visa holders in regions near San Francisco—San Francisco-Bay Area, North Coast, and Central Coast—scored the lowest in overall vulnerability.

[Table 3]

For peer review only - http://bmjopen.bmj.com/site/about/guidelines.xhtml

Table 3 presents the *concentration of vulnerability* for each immigrant status-region group. Groups whose vulnerability stems from low socioeconomic status are likely to share vulnerabilities from being a member of a minority group, experiencing language barriers (R=0.896), and having low access to health care (R=0.617). Groups' minority populations and language barriers are also correlated with high housing density (R=0.574) and low access to health care (R=0.761). High epidemiological and demographic vulnerabilities were not significantly correlated with high vulnerabilities from social causes. Table 4 presents a full correlation table with tests of statistical significance.

[Table 4]

Naturalized citizens had the fewest high-scoring (top 75th percentile) vulnerabilities with an average of 0.7 across ten regions. Undocumented immigrants had the most high-scoring vulnerabilities. Undocumented immigrants living in the Inland Empire scored in the top 75th percentile for five out of six vulnerability domains. Undocumented immigrants in other Southern Californian regions were similarly disadvantaged. They also had a high concentration of vulnerabilities (four out of six) even in regions where the vulnerability was low for other groups such as the Los Angeles and San Diego-Imperial areas.

Los Angeles County had the lowest concentration of vulnerability overall (an average of 0.8); no group aside undocumented immigrants had any high-scoring vulnerabilities. North Coast and the Central Coast regions had the highest concentration of vulnerability due to high scores among US-born natives and naturalized citizens in addition to immigrants with liminal statuses.

Discussion

Our study quantifies the degrees to which undocumented immigrants face disproportionate vulnerabilities during crises. Our domain-specific analyses showed that vulnerabilities from low socioeconomic status, language barriers, high housing density, and low access to health care go hand in hand and that these vulnerabilities are concentrated among undocumented immigrants living in

For peer review only - http://bmjopen.bmj.com/site/about/guidelines.xhtml

Southern California. The heightened social vulnerabilities among undocumented immigrants are not unique to COVID-19. Researchers have used the same factors to determine vulnerabilities in a wide range of crises, including the 2004 Tsunami in Aceh Indonesia [38] and Hurricane Katrina in New Orleans [37]. Our analysis of sensitive immigration status data in the CHIS demonstrates how much undocumented immigrants are marginalized and disadvantaged, even in a state that arguably has the most inclusive policies towards immigrants [18]. Despite undocumented immigrants' greater social vulnerabilities, demographic and COVID-19 specific epidemiological risk factors were the highest among U.S. citizens. These findings coincide with research that shows health advantages among recent immigrants that diminish to converge with US-born natives over time [39].

Our ecological approach also revealed regional disparities; San Joaquin Valleys and the Inland Empire had a high concentration of vulnerabilities among non-citizen immigrants. These regions may require parallel interventions to address the needs of a native population that is demographically and epidemiologically at-risk for COVID-19, as well as an immigrant population that is healthy but socioeconomically disadvantaged.

Researchers and policymakers should interpret the findings with caution. First, the data were collected in the years leading up to the COVID-19 pandemic. Thus, the vulnerabilities may diverge from the pandemic's peak in 2020 and 2021. Still, the structural inequities that we measure in our analysis have been profound and persistent in immigrant communities [19]. Furthermore, increased immigration policy restrictions and heightened enforcement in the past two years have brightened the divisions between legal immigration statuses[14]. Second, the vulnerability indices are relative measures among California's 50 immigrant status-region groups. Relative measures are more useful than absolute measures, however, when prioritizing groups and regions[29]. The domain-specific measures also do not compare across domains. It does not identify whether socioeconomic vulnerability matters more for COVID-19 outcomes than, say, having low health care access. In the absence of prior knowledge on these domains'

For peer review only - http://bmjopen.bmj.com/site/about/guidelines.xhtml

For peer review only

impact on COVID-19 outcomes, we have opted to place equal weight on each of the six domains. Third, the factors that we use in this study are associated with both the likelihood of infection (i.e., being a health care worker) and the likelihood of an adverse outcome upon infection (i.e., being obese). Some measures such as large household size incorporate both. Readers should consider the distinction depending on their aim.

Despite these limitations, this article concretely quantifies immigrants' unique and diverse vulnerabilities during the COVID-19 pandemic. Population-representative analysis of undocumented immigrants by sub-region is scarce, and our analysis aims to inform future disaster preparations.

Conclusion

Exclusionary policies against immigrants have created a nation that stratifies its people based on legal immigration status[40]. Immigrants are weaved into society as family members, neighbors, and coworkers of U.S.-born natives, and the consequences of ineffective public health measures among marginalized immigrants will spill over to everyone in the community [22,41]. In the absence of broad reform at the federal level, state and local governments must address the unique challenges immigrants face in their communities. Vaccination programs must explicitly engage with immigrants who have tenuous ties with the health care system and are wary of interactions with the government. Safety-net programs must be inclusive to all and actively overcome immigrants' reluctance to apply and enroll.

For peer review only - http://bmjopen.bmj.com/site/about/guidelines.xhtml

References

1. Langellier BA. Policy Recommendations to Address High Risk of COVID-19 Among Immigrants. *Am J Public Health*. 2020;110(8):1137-1139. doi:10.2105/AJPH.2020.305792

2. Hall M, Greenman E. The Occupational Cost of Being Illegal in the United States: Legal Status, Job Hazards, and Compensating Differentials. *Int Migr Rev*. 2015;49(2):406-442. doi:10.1111/imre.12090

3. Orrenius PM, Zavodny M. Help Wanted: Employer Demand for Less-Skilled Temporary Foreign Worker Visas in an Era of Declining Unauthorized Immigration. *RSF Russell Sage Found J Soc Sci*. 2020;6(3):45-67. doi:10.7758/RSF.2020.6.3.03

4. Van Hook J, Glick JE. Immigration and Living Arrangements: Moving Beyond Economic Need Versus Acculturation. *Demography*. 2007;44(2):225-249. doi:10.1353/dem.2007.0019

5. Phelan JC, Link BG, Tehranifar P. Social Conditions as Fundamental Causes of Health Inequalities: Theory, Evidence, and Policy Implications. *J Health Soc Behav*. 2010;51(1_suppl):S28-S40. doi:10.1177/0022146510383498

6. Asad AL, Clair M. Racialized legal status as a social determinant of health. *Soc Sci Med*. 2018;199:19-28. doi:10.1016/j.socscimed.2017.03.010

7. Hall M. Residential Integration on the New Frontier: Immigrant Segregation in Established and New Destinations. *Demography*. 2013;50(5):1873-1896. doi:http://dx.doi.org.proxy.library.emory.edu/10.1007/s13524-012-0177-x

8. Bacong A, Sohn H. Disentangling contributions of demographic, family, and socioeconomic factors on associations of immigration status and health in the United States. *J Epidemiol Community Health*. Published online November 25, 2020:jech-2020-214245. doi:10.1136/jech-2020-214245

9. Gelatt J. Do Employer-Sponsored Immigrants Fare Better in Labor Markets Than Family-Sponsored Immigrants? *RSF Russell Sage Found J Soc Sci*. 2020;6(3):70-93. doi:10.7758/RSF.2020.6.3.04

10. Perreira KM, Pedroza JM. Policies of Exclusion: Implications for the Health of Immigrants and Their Children. *Annu Rev Public Health*. 2019;40(1):147-166. doi:10.1146/annurev-publhealth-040218-044115

11. Donato KM, Amuedo-Dorantes C. The Legal Landscape of U.S. Immigration: An Introduction. *RSF Russell Sage Found J Soc Sci*. 2020;6(3):1-16. doi:10.7758/RSF.2020.6.3.01

12. Castañeda H, Holmes SM, Madrigal DS, Young M-ED, Beyeler N, Quesada J. Immigration as a Social Determinant of Health. *Annu Rev Public Health*. 2015;36(1):375-392. doi:10.1146/annurev-publhealth-032013-182419

13. Garcini LM, Domenech Rodríguez MM, Mercado A, Paris M. A tale of two crises: The compounded effect of COVID-19 and anti-immigration policy in the United States. *Psychol Trauma Theory Res Pract Policy*. 2020;12(S1):S230-S232. doi:10.1037/tra0000775

For peer review only - http://bmjopen.bmj.com/site/about/guidelines.xhtml

14. Zallman L, Finnegan KE, Himmelstein DU, Touw S, Woolhandler S. Implications of Changing Public Charge Immigration Rules for Children Who Need Medical Care. 2019;173. doi:10.1001/jamapediatrics.2019.1744

15. U.S. Citizenship and Immigration Services. *Inadmissibility on Public Charge Grounds*.; 2019. https://www.federalregister.gov/documents/2019/08/14/2019-17142/inadmissibility-on-public-charge-grounds

16. Motomura H. *Americans in Waiting: The Lost Story of Immigration and Citizenship in the United States*. Oxford University Press; 2006.

17. Huang X, Liu CY. Welcoming Cities: Immigration Policy at the Local Government Level. *Urban Aff Rev*. 2018;54(1):3-32. doi:10.1177/1078087416678999

18. Rodríguez MA, Young M-E, Wallace SP. *Creating Conditions to Support Healthy People: State Policies That Affect the Health of Undocumented Immigrants and Their Families*. University of California Global Health Institute; 2015:25.

19. De Trinidad Young M-E, León-Pérez G, Wells CR, Wallace SP. More Inclusive States, Less Poverty Among Immigrants? An Examination of Poverty, Citizenship Stratification, and State Immigrant Policies. *Popul Res Policy Rev*. 2018;37(2):205-228. doi:10.1007/s11113-018-9459-3

20. Martinez O, Wu E, Sandfort T, et al. Evaluating the Impact of Immigration Policies on Health Status Among Undocumented Immigrants: A Systematic Review. *J Immigr Minor Health*. 2015;17(3):947-970. doi:10.1007/s10903-013-9968-4

21. Atfeh M, Duperrault J, Wejsa S. A Dream Deferred: The Devastating Consequences of Restricting Undocumented Student Access to Higher Education in Georgia. Published online 2018:76.

22. Philbin MM, Flake M, Hatzenbuehler ML, Hirsch JS. State-level immigration and immigrant-focused policies as drivers of Latino health disparities in the United States. *Soc Sci Med*. 2018;199:29-38. doi:10.1016/j.socscimed.2017.04.007

23. Toomey RB, Umaña-Taylor AJ, Williams DR, Harvey-Mendoza E, Jahromi LB, Updegraff KA. Impact of Arizona's SB 1070 Immigration Law on Utilization of Health Care and Public Assistance Among Mexican-Origin Adolescent Mothers and Their Mother Figures. *Am J Public Health*. 2013;104(S1):S28-S34. doi:10.2105/AJPH.2013.301655

24. California Health Interview Survey. *CHIS 2014-2018 Adult Restricted Use Files.* UCLA Center for Health Policy Research; 2020.

25. Rendall MS, Ghosh-Dastidar B, Weden MM, Baker EH, Nazarov Z. Multiple Imputation for Combined-survey Estimation With Incomplete Regressors in One but Not Both Surveys. *Sociol Methods Res*. 2013;42(4):483-530. doi:10.1177/0049124113502947

26. Sohn H, Pebley A. New Approaches to Estimating Immigrant Documentation Status in Survey Data: *UCLA CCPR Popul Work Pap*. Published online June 26, 2020. Accessed December 14, 2020. http://128.97.186.17/index.php/pwp/article/view/1237

For peer review only - http://bmjopen.bmj.com/site/about/guidelines.xhtml

27. Passel J, Cohn D. *A Portrait of Unauthorized Immigrants in the United States*.; 2009:1-51. http://www.pewhispanic.org/files/reports/107.pdf

28. Centers for Disease Control and Prevention. CDC Social Vulnerability Index (SVI). Published October 15, 2020. Accessed December 17, 2020. https://www.atsdr.cdc.gov/placeandhealth/svi/index.html

29. Acharya R, Porwal A. A vulnerability index for the management of and response to the COVID-19 epidemic in India: an ecological study. *Lancet Glob Health*. 2020;8(9):e1142-e1151. doi:10.1016/S2214-109X(20)30300-4

30. Koma W, Artiga S, Neuman T, Claxton G. Low-Income and Communities of Color at Higher Risk of Serious Illness if Infected with Coronavirus. KFF. Published May 7, 2020. Accessed December 17, 2020. https://www.kff.org/coronavirus-covid-19/issue-brief/low-income-and-communities-of-color-at-higher-risk-of-serious-illness-if-infected-with-coronavirus/

31. Blackwell D, Lucas J, Clarke T. Summary Health Statistics for U.S. Adults: National Health Interview Survey, 2012. Published online 2014. doi:10.1037/e403882008-001

32. Rozenfeld Y, Beam J, Maier H, et al. A model of disparities: risk factors associated with COVID-19 infection. *Int J Equity Health*. 2020;19(1):126. doi:10.1186/s12939-020-01242-z

33. CDC. Coronavirus Disease 2019 (COVID-19). Centers for Disease Control and Prevention. Published February 11, 2020. Accessed December 21, 2020. https://www.cdc.gov/coronavirus/2019-ncov/need-extra-precautions/index.html

34. Migration Policy Institute. State Demographics Data. https://www.migrationpolicy.org/data/state-profiles/state/demographics/CA

35. Institute of Medicine. *Unequal Treatment: Confronting Racial and Ethnic Disparities in Health Care*. National Academies Press (US); 2003. doi:10.17226/12875

36. Chen JTS, Krieger N. Revealing the Unequal Burden of COVID-19 by Income, Race/Ethnicity, and Household Crowding: US County Versus Zip Code Analyses. *J Public Health Manag*. Published online February 2021. doi:10.1097/PHH.0000000000001263

37. Flanagan BE, Gregory EW, Hallisey EJ, Heitgerd JL, Lewis B. A Social Vulnerability Index for Disaster Management. *J Homel Secur Emerg Manag*. 2011;8(1). doi:10.2202/1547-7355.1792

38. Doocy S, Gorokhovich Y, Burnham G, Balk D, Robinson C. Tsunami mortality estimates and vulnerability mapping in Aceh, Indonesia. *Am J Public Health*. 2007;97 Suppl 1(Supplement_1):S146-51. doi:10.2105/AJPH.2006.095240

39. Antecol H, Bedard K. Unhealthy assimilation: Why do immigrants converge to American health status levels? *Demography*. 2006;43(2):337-360. doi:10.1353/dem.2006.0011

40. Massey DS. Creating the exclusionist society: from the War on Poverty to the war on immigrants. *Ethn Racial Stud*. 2020;43(1):18-37. doi:10.1080/01419870.2019.1667504

For peer review only - http://bmjopen.bmj.com/site/about/guidelines.xhtml

41. Pauly MV, Pagán JA. Spillovers and vulnerability: the case of community uninsurance. *Health Aff Proj Hope*. 2007;26(5):1304-1314. doi:10.1377/hlthaff.26.5.1304

For peer review only

For peer review only - http://bmjopen.bmj.com/site/about/guidelines.xhtml

Exhibit List

Exhibit 1 (table)

Caption: COVID-19 Social Vulnerability Index (SVI) domains and factors

Notes:

a Adapted from CDC's Social Vulnerability Index (CDC SVI)

b Adapted from Acharya and Porwal (2020)

c Adapted from Sugo Foundation's COVID-19 Community Vulnerability Index (CCVI)

d Author included

Exhibit 2 (table)

Caption: Domain-specific and overall social and COVID-19 vulnerability indices by California census region and immigrant status group

Source: Authors' analysis of the restricted data from the California Health Interview Survey (2014-2019).

Notes:

Values are vulnerability indices range from 1 (most vulnerable) to 0 (least vulnerable) within each domain. Vulnerability indices scoring above the 75th percentile (0.75) are highlighted in grey. Sample is limited to adults aged 18 and over. Distinction between documented visa holder and undocumented immigrants for years 2014, 2017, 2018, and 2019 is derived from multiple imputation using complete data in years 2015 and 2016.

a California's 2020 Census regions. Source: https://census.ca.gov/regions/

b Ranking is based on overall vulnerability. The ten most vulnerable groups are bolded.

Exhibit 3 (table)

Caption: Concentration of relative social and COVID-19 vulnerability by immigrant status and California Census region

Source: Authors' analysis of the restricted data from the California Health Interview Survey (2014-2019).

Notes:

Values indicate the number of vulnerability themes scoring in the top 75th percentile across 50 nativity/immigration status-region groups. Higher numbers indicate higher relative vulnerability. The maximum possible value is six. Sample is limited to adults aged 18 and over.

a California's 2020 Census regions. Source: https://census.ca.gov/regions/

For peer review only - http://bmjopen.bmj.com/site/about/guidelines.xhtml

b Unweighted average of the number of vulnerability themes scoring in the top 75th percentile

c Distinction between documented visa holder and undocumented immigrants for years 2014, 2017, 2018, and 2019 is derived from multiple imputation using complete data in years 2015 and 2016.

Exhibit 4 (table)

Caption: Correlation of vulnerability themes for nativity/immigration status-region groups

Source: Authors' analysis of the restricted data from the California Health Interview Survey (2014-2019).

Notes:

Correlations are calculated on vulnerability indices presented in Table 2. Only values significant at the $p < 0.005$ level are reported.

For peer review only

For peer review only - http://bmjopen.bmj.com/site/about/guidelines.xhtml

Table 1. COVID-19 Social Vulnerability Index (SVI) domains and factors

Domain		Factors	Description
1	**Socioeconomic Vulnerability [a]**	Below Poverty Level[a]	Calculated as the proportion of households at 0-99% Federal Poverty Level
		Unemployed[a]	Calculated as the proportion of households with both respondent and spouse (if present) unemployed
		No High School Diploma[a]	Calculated as the proportion of respondents with less than a high school diploma
2	**Demographic Vulnerability & Disability [a]**	Aged 65 or Older[a]	Calculated as the proportion of respondents aged 65 or older
		Single-Parent Household[a]	Calculated as the proportion of single parent households with children under 18 years old
		Psychological Disability[d]	Calculated as the proportion of respondents with a score of 13 or above on the Kessler Psychological Distress Scale
3	**Minority Status & Language Barriers [a]**	Minority[a]	Calculated as the proportion of non-White race or Hispanic ethnicity respondents
		Non-English Speaker[a]	Calculated as the proportion of respondents who speak English "not well" or "not at all"
4	**High Housing Density [a]**	Multi-Unit Structures / Mobile Homes[a]	Calculated as the proportion of respondents who live in a multi-family or mobile house
		Urbanization[b]	Calculated as the proportion of respondents who live in an urban or metropolitan area
		Extended Household[d]	Calculated as the proportion of households with three or more adults
5	**Epidemiological Risk Factors [c]**	High Blood Pressure[c]	Calculated as the proportion of respondents with ever physician-diagnosed high blood pressure
		Heart Disease[c]	Calculated as the proportion of respondents with ever physician-diagnosed heart disease
		Asthma[c]	Calculated as the proportion of respondents who reported currently having asthma
		Smoking[c]	Calculated as the proportion of respondents who reported being a current or former smoker
		Obesity[c]	Calculated as the proportion of respondents with a BMI of 30 or more for non-Asians or 27 or more for Asians
		Diabetes[c]	Calculated as the proportion of respondents with ever physician-diagnosed diabetes
		Heath Care Occupation [d]	Calculated as the proportion of respondents with an occupation in health care delivery
		High Risk Occupation [d]	Calculated as the proportion of respondents in essential occupations that have high risk of exposure to infectious diseases
6	**Low Access to Health Care [b]**	No Health Insurance[d]	Calculated as the proportion of respondents who reported having no health insurance in the past twelve months
		No Usual Source of Health Care [d]	Calculated as the proportion of respondents who reported no usual source of healthcare (i.e. doctor's office, community or government clinic, community hospital)

For peer review only - http://bmjopen.bmj.com/site/about/guidelines.xhtml

Notes

a Adapted from CDC's Social Vulnerability Index (CDC SVI)

b Adapted from Acharya and Porwal (2020)

c Adapted from Sugo Foundation's COVID-19 Community Vulnerability Index (CCVI)

d Author included

For peer review only

Table 2. Domain-specific and overall social and COVID-19 vulnerability indices by California census region and immigrant status group

Region[a]	Immigrant Status	Socioeconomic Vulnerability	Demographic Vulnerability & Disability	Minority Status & Language Barriers	High Housing Density	Epidemiological Risk Factors	Low Access to Health Care	Overall Vulnerability	Rank[b]
**Superior California**	US-born natives	0.163	0.918	0.020	0.061	0.816	0.143	0.102	45
	Naturalized citizens	0.327	0.612	0.224	0.102	0.673	0.041	0.245	38
	Legal permanent residents (LPR)	0.694	0.490	0.592	0.163	0.592	0.490	0.551	23
	Documented temporary visa holders	0.347	0.020	0.265	0.551	0.020	0.918	0.306	35
	Undocumented immigrants	0.837	0.122	0.857	0.265	1.000	0.898	0.918	**5**
**North Coast**	US-born natives	0.122	1.000	0.000	0.000	0.776	0.122	0.000	50
	Naturalized citizens	0.449	0.959	0.204	0.020	0.918	0.000	0.265	37
	Legal permanent residents (LPR)	0.653	0.653	0.673	0.531	0.980	0.388	0.694	16
	Documented temporary visa holders	0.592	0.224	0.408	0.347	0.082	0.633	0.347	33
	Undocumented immigrants	0.918	0.245	0.796	0.429	0.735	0.694	0.816	**10**
**San Francisco - Bay Area**	US-born natives	0.041	0.694	0.122	0.245	0.429	0.061	0.061	47
	Naturalized citizens	0.204	0.673	0.306	0.367	0.245	0.020	0.224	39
	Legal permanent residents (LPR)	0.265	0.265	0.571	0.653	0.265	0.408	0.388	31
	Documented temporary visa holders	0.000	0.000	0.245	0.673	0.000	0.429	0.020	49
	Undocumented immigrants	0.796	0.408	0.898	0.898	0.612	0.816	0.898	**6**
**Northern San Joaquin Valley**	US-born natives	0.245	0.939	0.102	0.041	0.959	0.347	0.143	43
	Naturalized citizens	0.673	0.755	0.367	0.082	0.490	0.204	0.367	32
	Legal permanent residents (LPR)	0.755	0.204	0.776	0.327	0.755	0.469	0.714	15
	Documented temporary visa holders	0.776	0.082	0.612	0.939	0.041	0.653	0.653	18
	Undocumented immigrants	0.878	0.388	0.959	0.694	0.327	0.735	0.837	**9**
**Central Coast**	US-born natives	0.061	0.878	0.041	0.184	0.449	0.102	0.041	48
	Naturalized citizens	0.367	0.571	0.347	0.388	0.367	0.163	0.286	36
	Legal permanent residents (LPR)	0.633	0.551	0.735	0.714	0.306	0.551	0.673	17
	Documented temporary visa holders	0.429	0.429	0.510	0.755	0.122	0.673	0.531	24
	Undocumented immigrants	0.857	0.184	1.000	0.735	0.184	0.980	0.878	**7**
**Southern San Joaquin Valley**	US-born natives	0.286	0.980	0.143	0.122	0.939	0.245	0.204	40
	Naturalized citizens	0.551	0.633	0.490	0.204	0.898	0.449	0.510	25
	Legal permanent residents (LPR)	0.816	0.469	0.816	0.510	0.286	0.510	0.776	12
	Documented temporary visa holders	0.531	0.041	0.694	0.571	0.163	0.837	0.612	20
	Undocumented immigrants	0.980	0.327	0.939	0.449	0.653	0.796	0.857	**8**
**Inland Empire**	US-born natives	0.224	0.857	0.163	0.224	0.694	0.367	0.184	41
	Naturalized citizens	0.571	0.735	0.449	0.306	0.857	0.286	0.490	26
	Legal permanent residents (LPR)	0.735	0.286	0.755	0.408	0.878	0.714	0.796	11
	Documented temporary visa holders	0.184	0.102	0.551	0.143	0.837	1.000	0.633	19
	Undocumented immigrants	1.000	0.510	0.918	0.837	0.347	0.857	0.939	**4**

For peer review only - http://bmjopen.bmj.com/site/about/guidelines.xhtml

**Los Angeles County**	US-born natives	0.143	0.837	0.184	0.469	0.469	0.327	0.163	42
	Naturalized citizens	0.510	0.898	0.429	0.633	0.551	0.265	0.469	27
	Legal permanent residents (LPR)	0.612	0.449	0.714	0.776	0.571	0.592	0.735	14
	Documented temporary visa holders	0.408	0.061	0.531	0.878	0.143	0.776	0.571	22
	Undocumented immigrants	0.959	0.367	0.837	1.000	0.714	0.878	0.980	**2**
**Orange County**	US-born natives	0.020	0.776	0.061	0.490	0.204	0.224	0.082	46
	Naturalized citizens	0.306	0.714	0.388	0.612	0.224	0.082	0.327	34
	Legal permanent residents (LPR)	0.469	0.347	0.633	0.796	0.531	0.531	0.592	21
	Documented temporary visa holders	0.102	0.306	0.327	0.959	0.102	0.755	0.429	29
	Undocumented immigrants	0.939	0.531	0.980	0.980	0.408	0.959	1.000	**1**
**San Diego - Imperial**	US-born natives	0.082	0.796	0.082	0.286	0.388	0.184	0.122	44
	Naturalized citizens	0.388	0.816	0.286	0.592	0.633	0.306	0.408	30
	Legal permanent residents (LPR)	0.714	0.592	0.653	0.816	0.510	0.571	0.755	13
	Documented temporary visa holders	0.490	0.163	0.469	0.918	0.061	0.612	0.449	28
	Undocumented immigrants	0.898	0.143	0.878	0.857	0.796	0.939	0.959	**3**

Notes

Values are vulnerability indices range from 1 (most vulnerable) to 0 (least vulnerable) within each domain. Vulnerability indices scoring above the 75th percentile (0.75) are highlighted in grey. Sample is limited to adults aged 18 and over. Distinction between documented visa holder and undocumented immigrants for years 2014, 2017, 2018, and 2019 is derived from multiple imputation using complete data in years 2015 and 2016. Data source: Restricted Data from the California Health Interview Survey (2014-2019).

a California's 2020 Census regions. Source: https://census.ca.gov/regions/

b Ranking is based on overall vulnerability. The ten most vulnerable groups are bolded.

For peer review only

Table 3. Concentration of relative social and COVID-19 vulnerability by immigrant status and California Census region

**Region[a]**	US-born Natives	Naturalized Citizens	Legal Permanent Residents (LPR)	Documented Temporary Visa Holders[c]	Undocumented Immigrants[c]	**Region Average[b]**
Superior California	1	0	0	1	3	1.0
North Coast	2	1	2	1	4	2.0
San Francisco-Bay Area	2	1	1	1	4	1.8
Northern San Joaquin Valley	0	2	1	2	2	1.4
Central Coast	2	1	3	2	2	2.0
Southern San Joaquin Valley	2	0	1	1	4	1.6
Inland Empire	1	1	1	1	5	1.8
Los Angeles County	0	0	0	0	4	0.8
Orange County	1	1	2	2	3	1.8
San Diego-Imperial	1	0	0	2	4	1.4
**Immigrant status group average[b]**	1.2	0.7	1.1	1.3	3.5	1.6

Notes

Values indicate the number of vulnerability themes scoring in the top 75th percentile across 50 nativity/immigration status-region groups. Higher numbers indicate higher relative vulnerability. The maximum possible value is six. Sample is limited to adults aged 18 and over. Data source: Restricted Data from the California Health Interview Survey (2014-2019)

a California's 2020 Census regions. Source: https://census.ca.gov/regions/

b Unweighted average of the number of vulnerability themes scoring in the top 75th percentile

c Distinction between documented visa holder and undocumented immigrants for years 2014, 2017, 2018, and 2019 is derived from multiple imputation using complete data in years 2015 and 2016.

For peer review only - http://bmjopen.bmj.com/site/about/guidelines.xhtml

Table 4. Correlation of vulnerability themes for nativity/immigration status-region groups

	Socioeconomic Vulnerability	Demographic Vulnerability & Disability	Minority Status & Language Barriers	High Housing Density	Epidemiological Risk Factors	Low Access to Health Care
**Socioeconomic Vulnerability**	1.000					
**Demographic Vulnerability & Disability**	insig.at p < 0.005	1.000				
**Minority Status & Language Barriers**	0.896	-0.597	1.000			
**High Housing Density**	0.398	-0.523	0.574	1.000		
**Epidemiological Risk Factors**	insig.at p < 0.005	0.406	insig.at p < 0.005	-0.526	1.000	
**Low Access to Health Care**	0.617	-0.766	0.761	0.593	insig.at p < 0.005	1.000

Notes

Correlations are calculated on vulnerability indices presented in Table 2. Only values significant at the $p < 0.005$ level are reported. Data source: Restricted Data from the California Health Interview Survey (2014-2019).

For peer review only - http://bmjopen.bmj.com/site/about/guidelines.xhtml

BMJ Open

Geographic Variation in COVID-19 Vulnerability by Legal Immigration Status in California: a pre-pandemic cross-sectional study

Journal:	*BMJ Open*
Manuscript ID	bmjopen-2021-054331.R1
Article Type:	Original research
Date Submitted by the Author:	06-Dec-2021
Complete List of Authors:	Sohn, Heeju; Emory University, Sociology<br>Aqua, Jasmine; Emory University, Rollins School of Public Health, Epidemiology
**Primary Subject Heading**:	Public health
Secondary Subject Heading:	Sociology
Keywords:	COVID-19, Public health < INFECTIOUS DISEASES, Epidemiology < TROPICAL MEDICINE

SCHOLARONE™
Manuscripts

I, the Submitting Author has the right to grant and does grant on behalf of all authors of the Work (as defined in the below author licence), an exclusive licence and/or a non-exclusive licence for contributions from authors who are: i) UK Crown employees; ii) where BMJ has agreed a CC-BY licence shall apply, and/or iii) in accordance with the terms applicable for US Federal Government officers or employees acting as part of their official duties; on a worldwide, perpetual, irrevocable, royalty-free basis to BMJ Publishing Group Ltd ("BMJ") its licensees and where the relevant Journal is co-owned by BMJ to the co-owners of the Journal, to publish the Work in this journal and any other BMJ products and to exploit all rights, as set out in our licence.

The Submitting Author accepts and understands that any supply made under these terms is made by BMJ to the Submitting Author unless you are acting as an employee on behalf of your employer or a postgraduate student of an affiliated institution which is paying any applicable article publishing charge ("APC") for Open Access articles. Where the Submitting Author wishes to make the Work available on an Open Access basis (and intends to pay the relevant APC), the terms of reuse of such Open Access shall be governed by a Creative Commons licence – details of these licences and which Creative Commons licence will apply to this Work are set out in our licence referred to above.

Other than as permitted in any relevant BMJ Author's Self Archiving Policies, I confirm this Work has not been accepted for publication elsewhere, is not being considered for publication elsewhere and does not duplicate material already published. I confirm all authors consent to publication of this Work and authorise the granting of this licence.

For peer review only

For peer review only

Geographic Variation in COVID-19 Vulnerability by Legal Immigration Status in California: a pre-pandemic cross-sectional study

Heeju Sohn, Ph.D.*

Department of Sociology

Emory University

heeju.sohn@emory.edu

Jasmine Ko Aqua, M.P.H.

Department of Epidemiology

Rollins School of Public Health

Emory University

* Corresponding author

Funding Information

This project was supported by grant number R00HD096322 from the Eunice Kennedy Shriver National Institute of Child Health and Human Development (NICHD).

For peer review only - http://bmjopen.bmj.com/site/about/guidelines.xhtml

Geographic Variation in COVID-19 Vulnerability by Legal Immigration Status in California: a pre-pandemic cross-sectional study

For peer review only

For peer review only - http://bmjopen.bmj.com/site/about/guidelines.xhtml

Contributorship statement

Heeju Sohn acquired funding and access to the restricted data, led the conceptualization and the analysis, and contributed to writing and editing the manuscript. Jasmine Aqua contributed to the methodology, the literature view, and the writing and editing of the manuscript.

Data sharing statement

The restricted version of the California Health Interview Survey (CHIS) is available through the Data Access Center at UCLA's Center for Health Policy Research. https://healthpolicy.ucla.edu/

Ethics statement

The use of the data for this project has been approved by the UCLA South General IRB (IRB #11-002227).

Competing interest statement

No competing interest

For peer review only

For peer review only - http://bmjopen.bmj.com/site/about/guidelines.xhtml

Abstract

Objective: To quantify COVID-19 vulnerabilities for Californian residents by their legal immigration status and place of residence.

Design: Secondary data analysis of cross-sectional population-representative survey data

Data: All adult respondents in the restricted version of the California Health Interview Survey (2015-2020, n = 128,528)

Outcome measure: Relative Social Vulnerability Indices for COVID-19 by legal immigration status and Census region across six domains: socioeconomic vulnerability; demography and disability; minority status and language barriers; high housing density; epidemiological risk; and access to care.

Results: Undocumented immigrants living in Southern California's urban areas (Los Angeles, Orange, San Diego-Imperial) have exceptionally high vulnerabilities due to low socioeconomic status, high language barriers, high housing density, and low access to care. San Joaquin Valley is home to vulnerable immigrant groups and a US-born population with the highest demographic and epidemiological risk for severe COVID-19.

Conclusion: Interventions to mitigate public health crises must explicitly consider immigrants' dual disadvantage from social vulnerability and exclusionary state and federal safety-net policies.

Keywords: COVID-19 Vulnerability, Immigrants, United States

For peer review only

Strengths and limitations of this study

1. We adapted the CDC's Social Vulnerability Index (SVI) to quantify immigrants' vulnerability to COVID-19 by their legal immigration status and their geographic region of residence in California.
2. Our analysis used the California Health Interview Survey (2015-2020, n = 128,528), which contains direct measurements of immigrants' legal status as well as detailed socioeconomic and health information.
3. The data covers 2015 to 2020, and vulnerability indices may diverge from the pandemic's peaks in 2021.
4. Vulnerability indices are relative measures among California's 50 immigrant status-region groups and cannot be generalized to the broader national population.

For peer review only

For peer review only - http://bmjopen.bmj.com/site/about/guidelines.xhtml

The novel coronavirus, severe acute respiratory syndrome coronavirus 2 (SARS-CoV-2), has spread across all parts of the United States, exacerbating entrenched social and health inequalities in its wake. This article uses sensitive immigration and geographic information from the restricted data in the California Health Interview Survey (CHIS) to quantify underlying socioeconomic, demographic, and epidemiological vulnerabilities to COVID-19 by legal immigration status in California's ten census regions.

Prior research on immigrants' resources and health suggests that their vulnerability to COVID-19 may be higher than the US-born population [1]. Higher prevalence of health conditions such as obesity, asthma, and diabetes among socioeconomically disadvantaged groups [2,3] suggests that immigrants may also have a higher risk for severe COVID-19 outcomes [4]. Many immigrants work in occupations that required in-person work throughout the pandemic [5,6] and live in larger households making isolation difficult [7], which indeed became an issue as the pandemic progressed, as commentaries describe health officials frequently discovering up to ten workers sharing a two-bedroom apartment or several families living in one house, most sick by the time contact tracers were able to notify them [8]. At the same time, immigrants comprise a large and diverse group [9,10], in which some sub-groups have high levels of education and income, whereas other sub-groups have high rates of poverty and economic insecurity. Unequal distribution of health care resources across geographic regions and residential segregation may also contribute to inequities in COVID-19 mortality within immigrant communities [11]. Yet, systematic and precise information on immigrants' vulnerabilities is absent from policymaking due to the lack of detailed immigration information in population-representative health surveys.

As the COVID-19 pandemic progressed, commentaries and limited research described the unique difficulties and high risk for adverse COVID-19 outcomes that undocumented immigrants faced. Reports from hospital emergency rooms detailed inpatient teams struggling to communicate with Spanish-speaking patients using language lines and through layers of personal protective equipment [8]. In

For peer review only - http://bmjopen.bmj.com/site/about/guidelines.xhtml

addition to reports of language barriers, a cross-sectional survey of adult, Spanish-speaking, non-citizen Latinx immigrants found that a substantial percentage of participants would not identify an undocumented household member or coworker during contact tracing, believed that uninsured immigrants were limited to hospital emergency departments for COVID-19 testing or treatment, and agreed that using public COVID-19 testing and treatment services could jeopardize an individual's immigration prospects [12]. Reports also noted high COVID-19 case rates and numerous significant outbreaks in federal immigrant detention centers [13–15], as well as a fear that convention centers that served as COVID-19 treatment facilities were actually immigration detention centers [8].

In addition to the COVID-19 risk factors and other unique difficulties described above, undocumented immigrants also face greater structural barriers in accessing health care and safety-net programs [16]. Federal policies dictate immigrants' access to federally funded health care services and safety-net programs [17,18] based on their immigration status. The March 2020 Coronavirus Aid, Relief, and Economic Security Act explicitly barred undocumented immigrants from receiving direct federal financial relief, continuing the long-standing policy of barring undocumented immigrants from cash assistance [19]. Legally present visa-holders and immigrants who have permission to live and work in the U.S. also have limited access to resources they can turn to during crises. The 1996 Personal Responsibility and Work Opportunity Reconciliation Act restricted legal immigrants' eligibility for federally funded safety-net programs, [17] and U.S. Citizenship and Immigration Services (USCIS) issued guidance at the beginning of the pandemic that legal immigrants could be denied citizenship or permanent residency for receiving an expanded range of eligible public health care benefits [20,21]. Though USCIS issued an alert on March 14, 2020, that COVID-19 testing, treatment, preventive care, and vaccines (when available) would be exempt from the "public charge" admissibility assessment and stopped applying this Public Charge Final Rule on March 9, 2021 [22], communication and implementation of these changes remained unclear, and

For peer review only - http://bmjopen.bmj.com/site/about/guidelines.xhtml

many immigrants still believed that using public COVID-19 testing and treatment services could jeopardize their immigration prospects [12].

When COVID-19 vaccines became available to the general public, the Department of Homeland Security (DHS) released a statement that they fully support equal access to the vaccine for undocumented immigrants [23]. Despite this public statement, the majority of state public health websites do not explicitly mention this information and many reports emerged throughout 2021 of undocumented immigrants being asked to provide Social Security Numbers at vaccination sites or being turned away for not presenting a state-issued ID [24–26]. Many undocumented immigrants also occupy jobs without paid leave and have language barriers that can impact their comprehension of vaccine information and education, introducing additional structural barriers to vaccination.

Policies at the state and local levels can support inclusive public health programs and outreach to address their immigrant communities' specific needs [27,28]. Some localities have expanded health care services to undocumented immigrant children and low-income pregnant women, allowed for in-state tuition and financial aid for undocumented students, and issued government identification to all residents [29]. Inclusive policies facilitate schooling and employment [30] for vulnerable immigrant groups and have been linked to better health outcomes [31]. California became the first state to provide COVID-19 disaster relief assistance to undocumented adults who are ineligible for other forms of assistance, providing a one-time direct assistance payment of $500 (maximum of $1000 per household) [32]. This $75 million dollar disaster relief assistance program was estimated to reach 150,000 undocumented Californians through twelve immigrant-serving nonprofit organizations [32]. The demand for relief quickly overwhelmed the available resources, with reports of people unable to get through phone lines due to extremely high call volumes and equating the direct assistance to "winning the lottery" [33].

For peer review only - http://bmjopen.bmj.com/site/about/guidelines.xhtml

For peer review only

Conversely, exclusionary policies such as mandating the use of E-Verify, an electronic database of immigrants' work authorization, or barring states from issuing drivers' licenses, or granting college admission to undocumented immigrants aim to create obstacles for those who do not have legal status [29,34]. Localities that coordinate with immigration enforcement also deter many immigrants and their families from seeking help regardless of their citizenship status [35,36].

This article identifies opportunities for local- and community-level interventions that can address the immigrants' unique challenges. Fractured policies that stratify people by immigration status stymies efforts that aim to mitigate the effects of the COVID-19 pandemic for all.

Methods

Data Source

We used the 2015-2020 survey data from CHIS, a collaborative data collection between UCLA's Center for Health Policy Research, the California Department of Public Health, and the Department of Health Care Service [37]1/1/0001 12:00:00 AM. The CHIS is a large, annual random-digit telephone survey of public health and health care access issues in California and is one of few representative surveys of this scale that collected information on detailed immigrant documentation status uncommon in large-scale surveys. The survey aims to produce estimates for underrepresented immigrant subgroups and administers the questionnaire in Spanish, Vietnamese, Korean, Mandarin, Cantonese, and Tagalog in addition to English. The use of the data for this project was approved by UCLA's South General IRB (IRB #11-002227).

Study Population

For peer review only - http://bmjopen.bmj.com/site/about/guidelines.xhtml

Our analysis included 128,528 adult survey respondents 18 years or older and used individual weights to account for sampling design. The CHIS imputed missing values for almost all variables in their surveys using random selection or hot deck imputation used in Census-published datasets.

Patient and public Involvement statement

The article presents analyses of secondary survey data, and no patients were involved in the study.

Documentation Status

We categorized respondents by their nativity and legal immigration status: US-born citizens, naturalized citizens, legal permanent residents (LPR), documented temporary visa holders, and undocumented immigrants. The first three categories, which accounted for almost 97 percent of our analysis sample, were determined directly for the entire study period 2015-2020 through a series of citizenship and immigration questions. All respondents answered whether they were born in the United States. If the response was no, they indicated whether they were naturalized citizens. Respondents who were not U.S. citizens were then asked whether they were LPRs. Questions that can differentiate undocumented immigrants from documented temporary visa holders (non-LPRs) were only asked in 2015-2016. The large majority (98.4 %) of our analysis sample had direct information on immigration status, including whether they were undocumented or living in the U.S. on valid visas. The remaining 1.6 percent of our sample non-citizens who were not LPRs in the years 2017-2020 accounted for about half of non-citizens and non-LPRs. They may have had a valid visa to live in the U.S. (i.e., students and diplomats), but CHIS did not ask for specific details on visa status during those survey years. We used a multiple imputation procedure to differentiate the documented temporary visa holders from the likely undocumented based on the relationships between sociodemographic characteristics and documentation status derived from the complete survey years in 2015 and 2016 [38,39]. We included age, age-squared, sex, educational attainment, country of origin, family type, English proficiency, years lived in the U.S., federal poverty

For peer review only - http://bmjopen.bmj.com/site/about/guidelines.xhtml

level, and geographic location in our multiple imputation procedure[40]. The imputation method to differentiate undocumented immigrants from documented temporary visa holders has been applied in national surveys such as the Survey of Income and Program Participation [41]. It has also been applied to impute immigration status in a “recipient” survey (American Community Survey) using data from a “donor” survey that directly collected immigration information [42,43]. These approaches are an extension of multiple imputation methods that leverage the relationships between variables with missing and known characteristics [44].

COVID-19 Vulnerability Index

We adapted the validated U.S. Centers for Disease Control and Prevention (CDC) ’s Social Vulnerability Index (SVI)[45] to develop a COVID-19 vulnerability index. We modified the CDC SVI based on the variables available in our dataset and expanded the index to include additional factors critical to the COVID-19 pandemic [46]. The first four themes in our COVID-vulnerability index (socioeconomic, demographic/disability, minority and language, and housing density) are based on CDC’s SVI. We were not able to include two factors from CDC’s SVI—physical/mental/emotional disability status and vehicle ownership—as CHIS did not ask these questions during the study period. Instead, we included a factor that indicated serious psychological distress based on Kessler’s Psychological Distress Questionnaire[47]. We also added a factor variable indicating the proportion of respondents living in an urban area to augment the CDC’s housing density theme. In addition to the CDC’s SVI four original themes, our analysis utilizes CHIS’s detailed health questionnaire and examined two more themes: epidemiological factors and access to health care. Overall, we incorporated 21 factors across six domains in our COVID-19 vulnerability index. Table 1 lists the six themes and their factors.

[Table 1]

For peer review only - http://bmjopen.bmj.com/site/about/guidelines.xhtml

For peer review only

Domain 1 – Socioeconomic Vulnerability: This domain captures the disproportionate crisis vulnerability associated with economic disadvantage. Households living below the poverty line face increased COVID-19 vulnerability due to structural health inequities and disproportionate distribution of underlying comorbidities[48,49]. Individuals with higher educational attainment have greater access to and may better adapt to COVID-19 risk communications and health messaging[50].

Domain 2 - Demographic Vulnerability & Disability: This domain captures the increased danger that vulnerable demographic groups face in disaster situations such as the COVID-19 pandemic. Older adults are at greater risk of requiring hospitalization or dying if diagnosed with COVID-19, and single parents and individuals with disabilities may experience additional stressors of the pandemic [51]. The pandemic has been particularly challenging for single-parent households, where only one parent is available for multiple responsibilities that may include working extra shifts, caring for a sick family member, or supervising online schooling.

Domain 3 - Minority Status & Language Barriers: This domain captures minority and marginalized populations' disproportionate vulnerability. About 33 percent of US-born and 60 percent of the foreign-born population in California self-reported as non-white[52], and they may encounter more racialized discrimination in health care settings than their white counterparts [53]. Limited English proficiency can be a barrier to accessing health services and understanding COVID-19 health messaging; recent studies linked low English proficiency with an increased risk of COVID-19 [50].

Domain 4 - High Housing Density: We included density factors associated with an increased risk of SARS-CoV-2 transmission: the proportion of respondents who live in a multi-family or mobile house, the proportion of respondents who live in an urban or metropolitan area, and the proportion of households with three or more adults [54].

For peer review only - http://bmjopen.bmj.com/site/about/guidelines.xhtml

Domain 5 - Epidemiological Risk Factors: This domain captures the medical and epidemiologic risk factors associated with COVID-19 infection and its adverse outcomes. The medical risk factors for severe COVID-19 in this domain include cardiovascular conditions (high blood pressure and heart disease), respiratory conditions (asthma and smoking), obesity, and diabetes [51]. Epidemiologic risk factors included occupations with a high risk of COVID-19 exposure. We used the O* NET's Work Surveys to identify high-risk occupations and cross-referenced them with California's Executive Order N-33-20 that defined essential workers. We harmonized the occupation categories with CHIS and assigned occupations in healthcare, service, transportation, construction, and extraction in the high-risk category.

Domain 6 - Low Access to Health Care: This domain encapsulates the additional vulnerability that health care barriers, such as the lack of health insurance, add during a widespread health crisis. Concerns about the cost of testing and treatment and uncertainty around where to seek medical attention lead to delayed patient care and disrupt our ability to control epidemics [50].

We constructed a vulnerability index for each of the six domains by immigration status intersected with census region (5 immigrant groups x 10 regions = 50 immigrant-region groups). First, we estimated groups' proportions in the high vulnerability category for each of the 21 factors. Second, we averaged the proportions across factors within each domain. Third, we ranked immigrant status-region groups from the group with the highest proportion in the vulnerable category to the lowest. We then assigned a percentile rank using the following equation: Percentile Rank = (rank – 1)/(N-1) where N equals 50 and represents the total number of immigrant status-region groups. A higher percentile indicates greater relative vulnerability. Our approach is the same as the method used by Acharya and Porwal [46], and Flanagan and colleagues [55].

Results

For peer review only - http://bmjopen.bmj.com/site/about/guidelines.xhtml

Table 2 summarizes demographic and socioeconomic characteristics by immigration status across California. The values are weighted by population and largely reflect the profiles of people living in urban areas. Similar to previous state-wide studies, documented temporary visa holders tended to be younger and healthier than other immigrant groups. Naturalized citizens are older than other groups with more health conditions than other immigrants. At the same time, they are less likely to live in poverty or without health insurance.

[Table 2]

Table 3 reports vulnerability indices in six domains for five immigrant groups living in California's ten census regions. Indices range from 0 (least vulnerable) to 1 (most vulnerable) and represent the relative vulnerability within 50 immigrant-region groups.

[Table 3]

Undocumented immigrants have high vulnerability due to low socioeconomic status, the concentration of minorities and language barriers and low access to care across the entire state. Undocumented immigrants living in the San Joaquin Valleys have the highest socioeconomic vulnerability. In contrast, vulnerability due to minority status and language barriers is the highest among undocumented immigrants in San Diego County (0.98) and Central Coast (1.00).

Naturalized citizens and US-born citizens share similar vulnerability profiles across the ten regions, but unlike non-citizen immigrants, their sources of vulnerability are predominantly from demographic composition and disability. They also score high in vulnerability from epidemiological COVID-19 risk factors, especially in the North Coast and the San Joaquin Valleys.

The wide range of vulnerability indices across California's regions reflects documented temporary visa holders' socioeconomic and demographic diversity that was obscured in Table 2. Documented

For peer review only - http://bmjopen.bmj.com/site/about/guidelines.xhtml

temporary visa holders living in the San Francisco-Bay Area are among the least vulnerable—they are the most socioeconomically and demographically advantaged (indices of 0.0) with low epidemiological risk for COVID-19. Conversely, documented temporary visa holders living in Southern San Joaquin Valley have a high socioeconomic vulnerability and low access to health care.

Vulnerability due to high housing density is concentrated among non-citizen immigrants, including LPRs in Southern California—Los Angeles County, Orange County, and San Diego-Imperial—and is likely linked to high housing costs in these regions. San Joaquin Valley is home to vulnerable non-citizen immigrants, including LPRs, due to their low socioeconomic status, high minority populations, and language barriers.

Table 3 also reports the overall vulnerability that combines all six domains, and the last column in the table indicates its ranking among the 50 immigrant status-region groups. Undocumented immigrants living in Southern California (Los Angeles County, Orange County, and San Diego-Imperial regions) had the highest overall vulnerability. US-born citizens and documented temporary visa holders in regions near San Francisco—San Francisco-Bay Area, North Coast, and Central Coast—scored the lowest in overall vulnerability.

[Table 4]

Table 4 presents the *concentration of vulnerability* for each immigrant status-region group. The values in Table 4 indicate the number of vulnerability themes out of a possible six that scored in the top 75th percentile. Table 5 presents a full correlation table between the six themes with tests of statistical significance. Groups whose vulnerability stems from low socioeconomic status are likely to share vulnerabilities from being a member of a minority group, experiencing language barriers ($R=0.858$), and having low access to health care ($R=0.561$). Groups' minority populations and language barriers are also correlated with high housing density ($R=0.574$) and low access to health care ($R=0.757$). High

For peer review only - http://bmjopen.bmj.com/site/about/guidelines.xhtml

epidemiological and demographic vulnerabilities were not significantly correlated with high vulnerabilities from social causes.

[Table 5]

Naturalized citizens had the fewest high-scoring (top 75th percentile) vulnerabilities with an average of 0.7 across ten regions. Undocumented immigrants had the most high-scoring vulnerabilities. Undocumented immigrants living in urban centers surrounding San Francisco, Los Angeles, and San Diego scored in the top 75th percentile for five out of six vulnerability domains. They also had a high concentration of vulnerabilities (four out of six) in non-urban regions where the vulnerability was low for other groups such as Superior California. North Coast and the Inland Empire regions had a relatively high concentration of vulnerability due to high scores among US-born citizens and naturalized citizens in addition to immigrants with liminal statuses.

Discussion

Our study highlights the unequal social vulnerabilities between people with different legal immigration statuses across California during the years leading up to the COVID-19 pandemic. Our domain-specific analyses showed that vulnerabilities from low socioeconomic status, language barriers, high housing density, and low access to health care go hand in hand and that these vulnerabilities are concentrated among undocumented immigrants living in Southern California. The heightened social vulnerabilities among undocumented immigrants are not unique to COVID-19. Researchers have used the same factors to determine vulnerabilities in a wide range of crises, including the 2004 Tsunami in Aceh Indonesia [56] and Hurricane Katrina in New Orleans [55]. Our analysis of sensitive immigration status data in the CHIS demonstrates how much undocumented immigrants are marginalized and disadvantaged, even in a state that arguably has the most inclusive policies towards immigrants [29]. Despite undocumented immigrants' greater social vulnerabilities, demographic and COVID-19 specific epidemiological risk

For peer review only - http://bmjopen.bmj.com/site/about/guidelines.xhtml

factors were the highest among U.S. citizens. These findings coincide with research that shows health advantages among recent immigrants that diminish to converge with US-born citizens over time [57].

Our ecological approach also revealed regional disparities by immigration status. Such disparities may require parallel interventions to address the needs of a US-born population that is demographically and epidemiologically at-risk for COVID-19, as well as an immigrant population that is healthy but socioeconomically disadvantaged.

Researchers and policymakers should interpret the findings with caution. First, the data were collected aggregated across the years leading up to the COVID-19 pandemic. Thus, the vulnerabilities may diverge from the pandemic's peak during the winter of 2020-2021. Still, the structural inequities that we measure in our analysis have been profound and persistent in immigrant communities [30]. Furthermore, increased immigration policy restrictions and heightened enforcement in the past two years have brightened the divisions between legal immigration statuses[20]. Second, the vulnerability indices are relative measures among California's 50 immigrant status-region groups. Relative measures are more useful than absolute measures, however, when prioritizing groups and regions[46]. The domain-specific measures also do not compare across domains. It does not identify whether socioeconomic vulnerability matters more for COVID-19 outcomes than, say, having low health care access. In the absence of prior knowledge on these domains' impact on COVID-19 outcomes, we have opted to place equal weight on each of the six domains. Third, the factors that we use in this study are not unilaterally associated with adverse outcomes from infection and disease progression. Some factors such as having an occupation in health care delivery can be both detrimental (i.e., exposure to the virus) and protective (i.e., income source and earlier access to vaccines). The factors are also not independent and can be connected in opposing directions. For example, people without a usual source of health care may be less likely to be diagnosed with comorbid conditions or work in health care occupations.

For peer review only - http://bmjopen.bmj.com/site/about/guidelines.xhtml

Despite these limitations, this article concretely examines immigrants' unique and diverse vulnerabilities associated with the COVID-19 pandemic. Population-representative analysis of undocumented immigrants by sub-region is scarce, and our analysis aims to inform future disaster preparations.

Conclusion

Exclusionary policies against immigrants have created a nation that stratifies its people based on legal immigration status[58]. Immigrants are weaved into society as family members, neighbors, and coworkers of US-born citizens, and the consequences of ineffective public health measures among marginalized immigrants will spill over to everyone in the community [35,59]. In the absence of broad reform at the federal level, state and local governments must address the unique challenges immigrants face in their communities. Vaccination programs must explicitly engage with immigrants who have tenuous ties with the health care system and are wary of interactions with the government. Safety-net programs must be inclusive to all and actively overcome immigrants' reluctance to apply and enroll.

For peer review only

For peer review only - http://bmjopen.bmj.com/site/about/guidelines.xhtml

References

1. Langellier BA. Policy Recommendations to Address High Risk of COVID-19 Among Immigrants. *Am J Public Health*. 2020;110(8):1137-1139. doi:10.2105/AJPH.2020.305792

2. Phelan JC, Link BG, Tehranifar P. Social Conditions as Fundamental Causes of Health Inequalities: Theory, Evidence, and Policy Implications. *J Health Soc Behav*. 2010;51(1_suppl):S28-S40. doi:10.1177/0022146510383498

3. Asad AL, Clair M. Racialized legal status as a social determinant of health. *Soc Sci Med*. 2018;199:19-28. doi:10.1016/j.socscimed.2017.03.010

4. Clark E, Fredricks K, Woc-Colburn L, Bottazzi ME, Weatherhead J. Disproportionate impact of the COVID-19 pandemic on immigrant communities in the United States. *PLoS Negl Trop Dis*. 2020;14(7):e0008484. doi:10.1371/journal.pntd.0008484

5. Hall M, Greenman E. The Occupational Cost of Being Illegal in the United States: Legal Status, Job Hazards, and Compensating Differentials. *Int Migr Rev*. 2015;49(2):406-442. doi:10.1111/imre.12090

6. Orrenius PM, Zavodny M. Help Wanted: Employer Demand for Less-Skilled Temporary Foreign Worker Visas in an Era of Declining Unauthorized Immigration. *RSF Russell Sage Found J Soc Sci*. 2020;6(3):45-67. doi:10.7758/RSF.2020.6.3.03

7. Van Hook J, Glick JE. Immigration and Living Arrangements: Moving Beyond Economic Need Versus Acculturation. *Demography*. 2007;44(2):225-249. doi:10.1353/dem.2007.0019

8. Page KR, Flores-Miller A. Lessons We've Learned — Covid-19 and the Undocumented Latinx Community. *N Engl J Med*. 2020;0(0):null. doi:10.1056/NEJMp2024897

9. Bacong A, Sohn H. Disentangling contributions of demographic, family, and socioeconomic factors on associations of immigration status and health in the United States. *J Epidemiol Community Health*. Published online November 25, 2020:jech-2020-214245. doi:10.1136/jech-2020-214245

10. Gelatt J. Do Employer-Sponsored Immigrants Fare Better in Labor Markets Than Family-Sponsored Immigrants? *RSF Russell Sage Found J Soc Sci*. 2020;6(3):70-93. doi:10.7758/RSF.2020.6.3.04

11. Hall M. Residential Integration on the New Frontier: Immigrant Segregation in Established and New Destinations. *Demography*. 2013;50(5):1873-1896. doi:http://dx.doi.org.proxy.library.emory.edu/10.1007/s13524-012-0177-x

12. Galletly CL, Lechuga J, Dickson-Gomez JB, Glasman LR, McAuliffe TL, Espinoza-Madrigal I. Assessment of COVID-19–Related Immigration Concerns Among Latinx Immigrants in the US. *JAMA Netw Open*. 2021;4(7):e2117049. doi:10.1001/jamanetworkopen.2021.17049

13. Openshaw JJ, Travassos MA. COVID-19 Outbreaks in US Immigrant Detention Centers: The Urgent Need to Adopt CDC Guidelines for Prevention and Evaluation. *Clin Infect Dis*. 2021;72(1):153-154. doi:10.1093/cid/ciaa692

For peer review only - http://bmjopen.bmj.com/site/about/guidelines.xhtml

14. Casanova FO, Hamblett A, Brinkley-Rubinstein L, Nowotny KM. Epidemiology of Coronavirus Disease 2019 in US Immigration and Customs Enforcement Detention Facilities. *JAMA Netw Open*. 2021;4(1):e2034409. doi:10.1001/jamanetworkopen.2020.34409

15. Erfani P, Uppal N, Lee CH, Mishori R, Peeler KR. COVID-19 Testing and Cases in Immigration Detention Centers, April-August 2020. *JAMA*. 2021;325(2):182-184. doi:10.1001/jama.2020.21473

16. Castañeda H, Holmes SM, Madrigal DS, Young MED, Beyeler N, Quesada J. Immigration as a Social Determinant of Health. *Annu Rev Public Health*. 2015;36(1):375-392. doi:10.1146/annurev-publhealth-032013-182419

17. Perreira KM, Pedroza JM. Policies of Exclusion: Implications for the Health of Immigrants and Their Children. *Annu Rev Public Health*. 2019;40(1):147-166. doi:10.1146/annurev-publhealth-040218-044115

18. Donato KM, Amuedo-Dorantes C. The Legal Landscape of U.S. Immigration: An Introduction. *RSF Russell Sage Found J Soc Sci*. 2020;6(3):1-16. doi:10.7758/RSF.2020.6.3.01

19. Garcini LM, Domenech Rodríguez MM, Mercado A, Paris M. A tale of two crises: The compounded effect of COVID-19 and anti-immigration policy in the United States. *Psychol Trauma Theory Res Pract Policy*. 2020;12(S1):S230-S232. doi:10.1037/tra0000775

20. Zallman L, Finnegan KE, Himmelstein DU, Touw S, Woolhandler S. Implications of Changing Public Charge Immigration Rules for Children Who Need Medical Care. 2019;173. doi:10.1001/jamapediatrics.2019.1744

21. U.S. Citizenship and Immigration Services. *Inadmissibility on Public Charge Grounds*.; 2019. https://www.federalregister.gov/documents/2019/08/14/2019-17142/inadmissibility-on-public-charge-grounds

22. U.S. Citizenship and Immigration Services. Public Charge | USCIS. Published August 19, 2021. Accessed November 15, 2021. https://www.uscis.gov/green-card/green-card-processes-and-procedures/public-charge

23. Department of Homeland Security. DHS Statement on Equal Access to COVID-19 Vaccines and Vaccine Distribution Sites. Department of Homeland Security. Published February 1, 2021. Accessed November 15, 2021. https://www.dhs.gov/news/2021/02/01/dhs-statement-equal-access-covid-19-vaccines-and-vaccine-distribution-sites

24. United We Dream. A Letter: COVID-19 Vaccine Access for Undocumented Communities. Accessed November 15, 2021. https://unitedwedream.org/wp-content/uploads/2021/05/Letter-COVID-19-Vaccine-Access-for-Undocumented-Communities.pdf

25. Semotiuk AJ. Immigrants Slow To Get Covid-19 Vaccine As Impediments Block Their Access. Forbes. Accessed November 15, 2021. https://www.forbes.com/sites/andyjsemotiuk/2021/09/01/immigrants-slow-to-get-covid-19-vaccine-as-impediments-block-their-access/

For peer review only - http://bmjopen.bmj.com/site/about/guidelines.xhtml

26. Safarpour A, Stokes M, 2021. KFF COVID-19 Vaccine Monitor: COVID-19 Vaccine Access, Information, and Experiences Among Hispanic Adults in the U.S. KFF. Published May 13, 2021. Accessed November 15, 2021. https://www.kff.org/coronavirus-covid-19/poll-finding/kff-covid-19-vaccine-monitor-access-information-experiences-hispanic-adults/

27. Motomura H. *Americans in Waiting: The Lost Story of Immigration and Citizenship in the United States*. Oxford University Press; 2006.

28. Huang X, Liu CY. Welcoming Cities: Immigration Policy at the Local Government Level. *Urban Aff Rev*. 2018;54(1):3-32. doi:10.1177/1078087416678999

29. Rodríguez MA, Young ME, Wallace SP. *Creating Conditions to Support Healthy People: State Policies That Affect the Health of Undocumented Immigrants and Their Families*. University of California Global Health Institute; 2015:25.

30. De Trinidad Young ME, León-Pérez G, Wells CR, Wallace SP. More Inclusive States, Less Poverty Among Immigrants? An Examination of Poverty, Citizenship Stratification, and State Immigrant Policies. *Popul Res Policy Rev*. 2018;37(2):205-228. doi:10.1007/s11113-018-9459-3

31. Martinez O, Wu E, Sandfort T, et al. Evaluating the Impact of Immigration Policies on Health Status Among Undocumented Immigrants: A Systematic Review. *J Immigr Minor Health*. 2015;17(3):947-970. doi:10.1007/s10903-013-9968-4

32. California Department of Social Services. Disaster Relief Assistance for Immigrants. Accessed November 15, 2021. https://www.cdss.ca.gov/inforesources/immigration/covid-19-drai

33. Jordan M. California Offers $500 in Covid-19 Aid to Undocumented Immigrants. *The New York Times*. https://www.nytimes.com/2020/05/18/us/coronavirus-undocumented-california.html. Published May 18, 2020. Accessed November 15, 2021.

34. Atfeh M, Duperrault J, Wejsa S. A Dream Deferred: The Devastating Consequences of Restricting Undocumented Student Access to Higher Education in Georgia. Published online 2018:76.

35. Philbin MM, Flake M, Hatzenbuehler ML, Hirsch JS. State-level immigration and immigrant-focused policies as drivers of Latino health disparities in the United States. *Soc Sci Med*. 2018;199:29-38. doi:10.1016/j.socscimed.2017.04.007

36. Toomey RB, Umaña-Taylor AJ, Williams DR, Harvey-Mendoza E, Jahromi LB, Updegraff KA. Impact of Arizona's SB 1070 Immigration Law on Utilization of Health Care and Public Assistance Among Mexican-Origin Adolescent Mothers and Their Mother Figures. *Am J Public Health*. 2013;104(S1):S28-S34. doi:10.2105/AJPH.2013.301655

37. California Health Interview Survey. *CHIS 2014-2018 Adult Restricted Use Files.* UCLA Center for Health Policy Research; 2020.

38. Rendall MS, Ghosh-Dastidar B, Weden MM, Baker EH, Nazarov Z. Multiple Imputation for Combined-survey Estimation With Incomplete Regressors in One but Not Both Surveys. *Sociol Methods Res*. 2013;42(4):483-530. doi:10.1177/0049124113502947

For peer review only - http://bmjopen.bmj.com/site/about/guidelines.xhtml

39. Sohn H, Pebley A. New Approaches to Estimating Immigrant Documentation Status in Survey Data: *UCLA CCPR Popul Work Pap*. Published online June 26, 2020. Accessed December 14, 2020. http://128.97.186.17/index.php/pwp/article/view/1237

40. Passel J, Cohn D. *A Portrait of Unauthorized Immigrants in the United States*.; 2009:1-51. http://www.pewhispanic.org/files/reports/107.pdf

41. Bachmeier JD, Van Hook J, Bean FD. Can We Measure Immigrants' Legal Status? Lessons from Two U.S. Surveys. *Int Migr Rev*. 2014;48(2):538-566. doi:10.1111/imre.12059

42. Rendall MS, Ghosh-Dastidar B, Weden MM, Baker EH, Nazarov Z. Multiple Imputation For Combined-Survey Estimation With Incomplete Regressors In One But Not Both Surveys. *Sociol Methods Res*. 2013;42(4). doi:10.1177/0049124113502947

43. Van Hook J, Bachmeier JD, Coffman DL, Harel O. Can We Spin Straw Into Gold? An Evaluation of Immigrant Legal Status Imputation Approaches. *Demography*. 2015;52(1):329-354. doi:10.1007/s13524-014-0358-x

44. Allison PD. Multiple Imputation for Missing Data: A Cautionary Tale. *Sociol Methods Res*. 2000;28(3):301-309. doi:10.1177/0049124100028003003

45. Centers for Disease Control and Prevention. CDC Social Vulnerability Index (SVI). Published October 15, 2020. Accessed December 17, 2020. https://www.atsdr.cdc.gov/placeandhealth/svi/index.html

46. Acharya R, Porwal A. A vulnerability index for the management of and response to the COVID-19 epidemic in India: an ecological study. *Lancet Glob Health*. 2020;8(9):e1142-e1151. doi:10.1016/S2214-109X(20)30300-4

47. Kessler RC, Barker PR, Colpe LJ, et al. Screening for serious mental illness in the general population. *Arch Gen Psychiatry*. 2003;60(2):184-189. doi:10.1001/archpsyc.60.2.184

48. Koma W, Artiga S, Neuman T, Claxton G. Low-Income and Communities of Color at Higher Risk of Serious Illness if Infected with Coronavirus. KFF. Published May 7, 2020. Accessed December 17, 2020. https://www.kff.org/coronavirus-covid-19/issue-brief/low-income-and-communities-of-color-at-higher-risk-of-serious-illness-if-infected-with-coronavirus/

49. Blackwell D, Lucas J, Clarke T. Summary Health Statistics for U.S. Adults: National Health Interview Survey, 2012. Published online 2014. doi:10.1037/e403882008-001

50. Rozenfeld Y, Beam J, Maier H, et al. A model of disparities: risk factors associated with COVID-19 infection. *Int J Equity Health*. 2020;19(1):126. doi:10.1186/s12939-020-01242-z

51. CDC. Coronavirus Disease 2019 (COVID-19). Centers for Disease Control and Prevention. Published February 11, 2020. Accessed December 21, 2020. https://www.cdc.gov/coronavirus/2019-ncov/need-extra-precautions/index.html

52. Migration Policy Institute. State Demographics Data. https://www.migrationpolicy.org/data/state-profiles/state/demographics/CA

For peer review only - http://bmjopen.bmj.com/site/about/guidelines.xhtml

53. Institute of Medicine. *Unequal Treatment: Confronting Racial and Ethnic Disparities in Health Care*. National Academies Press (US); 2003. doi:10.17226/12875

54. Chen JTS, Krieger N. Revealing the Unequal Burden of COVID-19 by Income, Race/Ethnicity, and Household Crowding: US County Versus Zip Code Analyses. *J Public Health Manag*. Published online February 2021. doi:10.1097/PHH.0000000000001263

55. Flanagan BE, Gregory EW, Hallisey EJ, Heitgerd JL, Lewis B. A Social Vulnerability Index for Disaster Management. *J Homel Secur Emerg Manag*. 2011;8(1). doi:10.2202/1547-7355.1792

56. Doocy S, Gorokhovich Y, Burnham G, Balk D, Robinson C. Tsunami mortality estimates and vulnerability mapping in Aceh, Indonesia. *Am J Public Health*. 2007;97 Suppl 1(Supplement_1):S146-51. doi:10.2105/AJPH.2006.095240

57. Antecol H, Bedard K. Unhealthy assimilation: Why do immigrants converge to American health status levels? *Demography*. 2006;43(2):337-360. doi:10.1353/dem.2006.0011

58. Massey DS. Creating the exclusionist society: from the War on Poverty to the war on immigrants. *Ethn Racial Stud*. 2020;43(1):18-37. doi:10.1080/01419870.2019.1667504

59. Pauly MV, Pagán JA. Spillovers and vulnerability: the case of community uninsurance. *Health Aff Proj Hope*. 2007;26(5):1304-1314. doi:10.1377/hlthaff.26.5.1304

For peer review only

For peer review only - http://bmjopen.bmj.com/site/about/guidelines.xhtml

Exhibit List

Exhibit 1 (table)

Caption: COVID-19 Social Vulnerability Index (SVI) domains and factors

Notes:

a Adapted from CDC's Social Vulnerability Index (CDC SVI)

b Adapted from Acharya and Porwal (2020)

c Adapted from Sugo Foundation's COVID-19 Community Vulnerability Index (CCVI)

d Author included

Exhibit 2 (table)

Caption: Demographic and socioeconomic characteristics of California residents by legal immigration status (2015-2020)

Source: Authors' analysis of the restricted data from the California Health Interview Survey (2015-2020).

Notes:

Sample is limited to adults aged 18 and over. Distinction between documented temporary visa holders and undocumented immigrants for years 2017-2020 is derived from multiple imputation using complete data in years 2015 and 2016.

a Included in socioeconomic vulnerability domain

b Included in demographic vulnerability and disability domain

c Included in minority status and language barrier domain

d Included in high housing density domain

e Included in epidemiological risk domain

f Included in health care access domain

Exhibit 3 (table)

Caption: Domain-specific and overall social and COVID-19 vulnerability indices by California census region and immigrant status group

Source: Authors' analysis of the restricted data from the California Health Interview Survey (2015-2020).

Notes:

For peer review only - http://bmjopen.bmj.com/site/about/guidelines.xhtml

Values are vulnerability indices ranging from 1 (most vulnerable) to 0 (least vulnerable) within each domain.

Vulnerability indices scoring above the 75th percentile (0.75) are highlighted in grey. Sample is limited to adults aged 18 and over.

a California's 2020 Census regions. Source: https://census.ca.gov/regions/

b Ranking is based on overall vulnerability. The ten most vulnerable groups are bolded.

Exhibit 4 (table)

Caption: Concentration of relative social and COVID-19 vulnerability by immigrant status and California Census region

Source: Authors' analysis of the restricted data from the California Health Interview Survey (2015-2020).

Notes:

Values indicate the number of vulnerability themes scoring in the top 75th percentile across 50 nativity/immigration status-region groups. Higher numbers indicate higher relative vulnerability. The maximum possible value is six. Sample is limited to adults aged 18 and over.

a California's 2020 Census regions. Source: https://census.ca.gov/regions/

b Unweighted average of the number of vulnerability themes scoring in the top 75th percentile

c Distinction between documented temporary visa holders and undocumented immigrants for years 2017-2020 is derived from multiple imputation using complete data in years 2015 and 2016.

Exhibit 5 (table)

Caption: Correlation of vulnerability themes for nativity/immigration status-region groups

Source: Authors' analysis of the restricted data from the California Health Interview Survey (2015-2020).

Notes:

Correlations are calculated on vulnerability indices presented in Table 2. Only values significant at the $p < 0.005$ level are reported.

For peer review only - http://bmjopen.bmj.com/site/about/guidelines.xhtml

Table 1. COVID-19 Social Vulnerability Index (SVI) domains and factors

Domain		Factors	Description
1	Socioeconomic Vulnerability [a]	Below Poverty Level[a]	Calculated as the proportion of households at 0-99% Federal Poverty Level
		Unemployed[a]	Calculated as the proportion of households with both respondent and spouse (if present) unemployed
		No High School Diploma[a]	Calculated as the proportion of respondents with less than a high school diploma
2	Demographic Vulnerability & Disability [a]	Aged 65 or Older[a]	Calculated as the proportion of respondents aged 65 or older
		Single-Parent Household[a]	Calculated as the proportion of single parent households with children under 18 years old
		Psychological Disability[d]	Calculated as the proportion of respondents with a score of 13 or above on the Kessler Psychological Distress Scale
3	Minority Status & Language Barriers [a]	Minority[a]	Calculated as the proportion of non-White race or Hispanic ethnicity respondents
		Non-English Speaker[a]	Calculated as the proportion of respondents who speak English “not well” or “not at all”
4	High Housing Density [a]	Multi-Unit Structures / Mobile Homes[a]	Calculated as the proportion of respondents who live in a multi-family or mobile house
		Urbanization[b]	Calculated as the proportion of respondents who live in an urban or metropolitan area
		Extended Household[d]	Calculated as the proportion of households with three or more adults
5	Epidemiological Risk Factors [c]	High Blood Pressure[c]	Calculated as the proportion of respondents with ever physician-diagnosed high blood pressure
		Heart Disease[c]	Calculated as the proportion of respondents with ever physician-diagnosed heart disease
		Asthma[c]	Calculated as the proportion of respondents who reported currently having asthma
		Smoking[c]	Calculated as the proportion of respondents who reported being a current or former smoker
		Obesity[c]	Calculated as the proportion of respondents with a BMI of 30 or more for non-Asians or 27 or more for Asians
		Diabetes[c]	Calculated as the proportion of respondents with ever physician-diagnosed diabetes
		Heath Care Occupation [d]	Calculated as the proportion of respondents with an occupation in health care delivery
		High Risk Occupation [d]	Calculated as the proportion of respondents in essential occupations that have high risk of exposure to infectious diseases
6	Low Access to Health Care [b]	No Health Insurance[d]	Calculated as the proportion of respondents who reported having no health insurance in the past twelve months
		No Usual Source of Health Care [d]	Calculated as the proportion of respondents who reported no usual source of healthcare (i.e. doctor’s office, community or government clinic, community hospital)

For peer review only - http://bmjopen.bmj.com/site/about/guidelines.xhtml

Notes

a Adapted from CDC's Social Vulnerability Index (CDC SVI)

b Adapted from Acharya and Porwal (2020)

c Adapted from Surgo Foundation's COVID-19 Community Vulnerability Index (CCVI)

d Author included

For peer review only

For peer review only - http://bmjopen.bmj.com/site/about/guidelines.xhtml

Table 2. Demographic and socioeconomic characteristics of California residents by legal immigration status (2015-2020)

		US-born citizens<br>n = 100,387 (78.1%)	Naturalized citizens<br>n = 18,386 (14.3%)	Legal permanent residents (LPR)<br>n = 5,825 (4.5%)	Documented temporary visa holders<br>n = 2,813 (2.2%)	Undocumented immigrants<br>n = 1,117 (0.9%)
Demographic characteristics						
Mean age	b	46.2	52.5	45.6	33.9	38.9
Mean family size		2.1	2.3	2.6	2.3	3.0
Mean household size		3.1	3.5	3.8	3.1	4.5
Mean years lived in the US		na	31.4	20.0	7.6	16.7
Proportion female		0.51	0.53	0.50	0.48	0.47
Proportion non-white or Hispanic	c	0.45	0.85	0.90	0.86	0.99
Proportion in households with 3 or more adults	d	0.40	0.50	0.52	0.31	0.54
Proportion living in single-parent household	b	0.07	0.06	0.07	0.08	0.17
Proportion living in urban area	d	0.97	0.99	0.99	1.00	0.99
Socioeconomic characteristics						
Proportion with household incomes below 100 FPL	a	0.12	0.16	0.25	0.21	0.46
Proportions with no earners in family	a	0.30	0.27	0.19	0.17	0.16
Proportion without a HS degree or equivalent	a	0.07	0.26	0.44	0.11	0.64
Proportion living in a multi-unit structure or a mobile home	d	0.13	0.15	0.19	0.11	0.30
Proportion without health insurance	f	0.29	0.30	0.42	0.66	0.56
Proportion with no usual source of health care	f	0.06	0.07	0.13	0.20	0.42
Proportion in healthcare-related occupation	e	0.03	0.03	0.01	0.02	0.00
Proportion in occupations with close physical contact with others	e	0.15	0.14	0.26	0.33	0.41
Proportion who speaks English not well or not at all	c	0.01	0.26	0.49	0.20	0.72
Health characteristics						
Proportion with fair or poor self-rated health		0.16	0.24	0.29	0.11	0.34
Proportion scoring above the threshold for psychological distress in past 12 months	b	0.20	0.23	0.10	0.00	0.02
Proportion with at least one comorbid condition: asthma, diabetes, cardiovascular disease, high blood pressure, obese, current/former smoker	e	0.61	0.56	0.56	0.42	0.59

Source: Authors' analysis of the restricted data from the California Health Interview Survey (2015-2020).

Notes:

Sample is limited to adults aged 18 and over. Distinction between documented temporary visa holders and undocumented immigrants for years 2017-2020 is derived from multiple imputation using complete data in years 2015 and 2016.

a Included in socioeconomic vulnerability domain

b Included in demographic vulnerability and disability domain

c Included in minority status and language barrier domain

d Included in high housing density domain

e Included in epidemiological risk domain

f Included in health care access domain

For peer review only - http://bmjopen.bmj.com/site/about/guidelines.xhtml

Table 3. Domain-specific and overall social and COVID-19 vulnerability indices by California census region and immigrant status group

Region[a]	Immigrant Status	N (unweighted)	Socioeconomic Vulnerability	Demographic Vulnerability & Disability	Minority Status & Language Barriers	High Housing Density	Epidemiological Risk Factors	Low Access to Health Care	Overall Vulnerability	Rank[b]
**Superior California**	US-born citizens	16,588	0.163	0.898	0.020	0.020	0.735	0.122	0.041	48
	Naturalized citizens	1,357	0.429	0.612	0.265	0.122	0.449	0.184	0.265	37
	Legal permanent residents (LPR)	442	0.653	0.245	0.612	0.224	0.184	0.490	0.469	27
	Documented temporary visa holders	58	0.469	0.469	0.429	0.510	0.041	0.735	0.347	33
	Undocumented immigrants	183	0.878	0.163	0.796	0.571	0.837	0.898	0.857	**8**
**North Coast**	US-born citizens	7,455	0.122	1.000	0.000	0.000	0.857	0.143	0.000	50
	Naturalized citizens	472	0.490	0.980	0.224	0.041	0.918	0.041	0.306	35
	Legal permanent residents (LPR)	189	0.633	0.510	0.673	0.163	0.980	0.245	0.633	19
	Documented temporary visa holders	26	0.837	0.061	0.204	0.327	0.000	0.000	0.143	43
	Undocumented immigrants	97	0.796	0.122	0.878	0.449	1.000	0.776	0.878	**7**
**San Francisco - Bay Area**	US-born citizens	15,060	0.020	0.776	0.102	0.245	0.429	0.082	0.061	47
	Naturalized citizens	3,728	0.184	0.653	0.347	0.367	0.204	0.020	0.245	38
	Legal permanent residents (LPR)	949	0.265	0.204	0.551	0.673	0.265	0.449	0.408	30
	Documented temporary visa holders	400	0.000	0.000	0.245	0.694	0.020	0.510	0.020	49
	Undocumented immigrants	336	0.755	0.551	0.898	0.959	0.898	0.796	0.898	**6**
**Northern San Joaquin Valley**	US-born citizens	6,215	0.327	0.939	0.122	0.061	0.939	0.327	0.163	42
	Naturalized citizens	628	0.592	0.694	0.408	0.082	0.531	0.102	0.388	31
	Legal permanent residents (LPR)	341	0.776	0.265	0.755	0.306	0.551	0.571	0.714	15
	Documented temporary visa holders	16	0.510	0.020	0.653	0.592	0.082	0.673	0.449	28
	Undocumented immigrants	195	0.980	0.306	0.939	0.633	0.347	0.755	0.837	**9**
**Central Coast**	US-born citizens	7,859	0.061	0.878	0.041	0.184	0.388	0.204	0.082	46
	Naturalized citizens	997	0.388	0.673	0.327	0.469	0.367	0.224	0.367	32
	Legal permanent residents (LPR)	404	0.694	0.531	0.735	0.755	0.571	0.612	0.735	14
	Documented temporary visa holders	54	0.306	0.592	0.449	0.735	0.163	0.714	0.571	22
	Undocumented immigrants	275	0.939	0.102	1.000	0.796	0.612	0.980	0.939	**4**
**Southern San Joaquin Valley**	US-born citizens	6,386	0.347	0.959	0.143	0.102	0.959	0.347	0.224	39
	Naturalized citizens	724	0.612	0.755	0.510	0.143	0.755	0.388	0.490	26
	Legal permanent residents (LPR)	376	0.816	0.388	0.816	0.347	0.673	0.531	0.796	11
	Documented temporary visa holders	29	0.531	0.041	0.571	0.551	0.122	0.857	0.612	20
	Undocumented immigrants	292	1.000	0.286	0.959	0.429	0.224	0.837	0.816	**10**
**Inland Empire**	US-born citizens	8,068	0.204	0.918	0.163	0.204	0.816	0.367	0.204	40
	Naturalized citizens	1,313	0.571	0.796	0.531	0.286	0.796	0.429	0.592	21
	Legal permanent residents (LPR)	463	0.735	0.327	0.776	0.388	0.694	0.633	0.755	13
	Documented temporary visa holders	55	0.286	0.184	0.592	0.653	0.592	0.816	0.673	17
	Undocumented immigrants	210	0.959	0.490	0.837	0.776	0.490	0.959	0.918	**5**

For peer review only - http://bmjopen.bmj.com/site/about/guidelines.xhtml

**Los Angeles County**	US-born citizens	16,210	0.143	0.857	0.184	0.490	0.510	0.408	0.184	41
	Naturalized citizens	5,132	0.551	0.816	0.469	0.714	0.633	0.265	0.551	23
	Legal permanent residents (LPR)	1,477	0.714	0.408	0.714	0.878	0.714	0.592	0.776	12
	Documented temporary visa holders	297	0.449	0.082	0.490	0.837	0.102	0.694	0.510	25
	Undocumented immigrants	886	0.918	0.429	0.857	0.980	0.776	0.939	0.980	**2**
**Orange County**	US-born citizens	4,987	0.041	0.837	0.082	0.408	0.286	0.306	0.102	45
	Naturalized citizens	1,498	0.224	0.633	0.388	0.612	0.245	0.061	0.286	36
	Legal permanent residents (LPR)	315	0.408	0.143	0.633	0.857	0.327	0.469	0.531	24
	Documented temporary visa holders	75	0.245	0.367	0.306	0.918	0.143	0.878	0.653	18
	Undocumented immigrants	126	0.857	0.449	0.918	1.000	0.306	1.000	1.000	**1**
**San Diego - Imperial**	US-born citizens	11,559	0.082	0.735	0.061	0.265	0.408	0.163	0.122	44
	Naturalized citizens	2,537	0.367	0.714	0.286	0.531	0.653	0.286	0.429	29
	Legal permanent residents (LPR)	869	0.673	0.571	0.694	0.816	0.469	0.551	0.694	16
	Documented temporary visa holders	107	0.102	0.224	0.367	0.898	0.061	0.653	0.327	34
	Undocumented immigrants	213	0.898	0.347	0.980	0.939	0.878	0.918	0.959	**3**

Notes

Values are vulnerability indices range from 1 (most vulnerable) to 0 (least vulnerable) within each domain. Vulnerability indices scoring above the 75th percentile (0.75) are highlighted in grey. Sample is limited to adults aged 18 and over. Distinction between documented temporary visa holders and undocumented immigrants for years 2017-2020 is derived from multiple imputation using complete data in years 2015 and 2016. Data source: Restricted Data from the California Health Interview Survey (2015-2020).

a California's 2020 Census regions. Source: https://census.ca.gov/regions/

b Ranking is based on overall vulnerability. The ten most vulnerable groups are bolded.

For peer review only

For peer review only - http://bmjopen.bmj.com/site/about/guidelines.xhtml

Table 4. Concentration of relative social and COVID-19 vulnerability by immigrant status and California Census region

**Region[a]**	US-born Citizens	Naturalized Citizens	Legal Permanent Residents (LPR)	Documented Temporary Visa Holders[c]	Undocumented Immigrants[c]	**Region Average[b]**
Superior California	1	0	0	0	4	1.0
North Coast	2	2	1	1	4	2.0
San Francisco-Bay Area	1	0	0	0	5	1.2
Northern San Joaquin Valley	2	0	2	0	3	1.4
Central Coast	1	0	1	0	4	1.2
Southern San Joaquin Valley	2	2	2	1	3	2.0
Inland Empire	2	2	1	1	4	2.0
Los Angeles County	1	1	1	1	5	1.8
Orange County	1	0	1	2	4	1.6
San Diego-Imperial	0	0	1	1	5	1.4
**Immigrant status group average[b]**	1.3	0.7	1.0	0.7	4.1	1.6

Notes

Values indicate the number of vulnerability themes scoring in the top 75th percentile across 50 nativity/immigration status-region groups. Higher numbers indicate higher relative vulnerability. The maximum possible value is six. Sample is limited to adults aged 18 and over. Data source: Restricted Data from the California Health Interview Survey (2015-2020)

a California's 2020 Census regions. Source: https://census.ca.gov/regions/

b Unweighted average of the number of vulnerability themes scoring in the top 75th percentile

c Distinction between documented temporary visa holders and undocumented immigrants for years 2017-2020 is derived from multiple imputation using complete data in years 2015 and 2016.

For peer review only - http://bmjopen.bmj.com/site/about/guidelines.xhtml

Table 5. Correlation of vulnerability themes for nativity/immigration status-region groups

	Socioeconomic Vulnerability	Demographic Vulnerability & Disability	Minority Status & Language Barriers	High Housing Density	Epidemiological Risk Factors	Low Access to Health Care
**Socioeconomic Vulnerability**	1.000					
**Demographic Vulnerability & Disability**	-0.421	1.000				
**Minority Status & Language Barriers**	0.858	-0.603	1.000			
**High Housing Density**	insig.at p < 0.005	-0.542	0.574	1.000		
**Epidemiological Risk Factors**	insig.at p < 0.005	0.445	insig.at p < 0.005	insig.at p < 0.005	1.000	
**Low Access to Health Care**	0.561	-0.597	0.757	0.703	-0.052	1.000

Notes

Correlations are calculated on vulnerability indices presented in Table 2. Only values significant at the p < 0.005 level are reported. Data source: Restricted Data from the California Health Interview Survey (2015-2020).

For peer review only - http://bmjopen.bmj.com/site/about/guidelines.xhtml
